# High-integrity human intervention in ecosystems: Tracking self-organization modes

**DOI:** 10.1371/journal.pcbi.1009427

**Published:** 2021-09-29

**Authors:** Yuval R. Zelnik, Yair Mau, Moshe Shachak, Ehud Meron

**Affiliations:** 1 Centre for Biodiversity Theory and Modelling, Theoretical and Experimental Ecology Station, CNRS, Moulis, France; 2 Department of Ecology, Swedish University of Agricultural Sciences, Uppsala, Sweden; 3 The Institute of Environmental Sciences, The Robert H. Smith Faculty of Agriculture, Food and Environment, The Hebrew University of Jerusalem, Rehovot, Israel; 4 Mitrani Department of Desert Ecology, Blaustein Institutes for Desert Research, Ben-Gurion University of the Negev, Sede Boqer Campus, Israel; 5 Department of Solar Energy and Environmental physics, Blaustein Institutes for Desert Research, Ben-Gurion University of the Negev, Sede Boqer Campus, Israel; 6 Department of Physics, Ben-Gurion University of the Negev, Beer-Sheva, Israel; University of Zaragoza: Universidad de Zaragoza, SPAIN

## Abstract

Humans play major roles in shaping and transforming the ecology of Earth. Unlike natural drivers of ecosystem change, which are erratic and unpredictable, human intervention in ecosystems generally involves planning and management, but often results in detrimental outcomes. Using model studies and aerial-image analysis, we argue that the design of a successful human intervention form calls for the identification of the self-organization modes that drive ecosystem change, and for studying their dynamics. We demonstrate this approach with two examples: grazing management in drought-prone ecosystems, and rehabilitation of degraded vegetation by water harvesting. We show that grazing can increase the resilience to droughts, rather than imposing an additional stress, if managed in a spatially non-uniform manner, and that fragmental restoration along contour bunds is more resilient than the common practice of continuous restoration in vegetation stripes. We conclude by discussing the need for additional studies of self-organization modes and their dynamics.

## Author summary

Human intervention in ecosystems is motivated by various functional needs, such as provisioning ecosystem services, but often has unexpected detrimental outcomes. A major question in ecology is how to manage human intervention so as to achieve its goal without impairing ecosystem function. The main idea pursued here is the need to identify the inherent response ways of ecosystems to disturbances, and use them as road maps for conducting interventions. This approach is demonstrated mathematically using two contexts, grazing management and vegetation restoration, and compared to remote sensing data for the latter. Among the surprising insights obtained is the beneficial effect of grazing, in terms of resilience to droughts, that can be achieved by managing it non-uniformly in space.

## Introduction

The dominant role played by humans in shaping and transforming the ecology of the Earth is well recognized [1]. Human intervention in ecosystems is driven by various needs, including ecosystem services, land-use changes, and rehabilitation of degraded ecosystems [2]. Unlike unpredictable natural drivers of ecosystem change, such as droughts, forest fires and pest outbreaks, human drivers typically involve planning and controlled management. Yet, the outcomes of human intervention are often detrimental to the ecosystems involved, often when coupled to natural drivers [3]; biodiversity loss [4], desertification [5, 6], and degradation of coastal ecosystems [7], are a few examples of such detrimental human impact. Planning human intervention under conditions of environmental variability, without impairing ecosystem function, is thus a major challenge of current ecological research.

An instrumental concept that emerged in the context of human intervention is that of ‘ecological integrity’, viewed here as a system attribute that reflects the degree to which an ecosystem is self-organized in a functional ecosystem state [8, 9]. Implicit in this concept are three premises. i) Ecosystems are nonlinear dynamical systems that approach a *stable state* (see short glossary in Table 1 and detailed glossary in S1 Appendix) when left undisturbed [10]. ii) The approach to a functional stable state, i.e. a state of a steadily functioning ecosystem, is a self-organization process, which involves the emergence of spatially heterogeneous landscapes, networks of energy and resource flows, complex food webs, and high species diversity [8, 11, 12]. iii) Human intervention often interferes with these self-organization processes, typically resulting in reduced functionality and ecological integrity [13]. The introduction of the integrity concept is highly constructive in focusing attention on the natural tendency of ecosystems to self-organize. However, most efforts have been devoted to the intricate question [3] of assessing ecological integrity [8, 9], overlooking the dynamics of self-organization and the implications of these dynamics to management practices.

**Table 1:** A short glossary, defining the central terms used in this manuscript. See S1 Appendix for more detailed glossary, with more detailed definitions and a more comprehensive list of terms.

Main terms	Short definitions
Basin of attraction	The set of initial conditions that converge in time to the same stable state.
Bifurcation diagram	A diagram that shows the existence ranges and stability properties of possible systems states.
Growing eigenmode	The direction in phase space along which a system changes following an instability.
Instability (bifurcation)	A threshold phenomenon where small perturbations of a system state are amplified along a particular eigenmode and induce a state change.
Linear stability analysis	A mathematical method to identify instabilities of systems states by analyzing the dynamics of diminishingly small perturbations.
Phase space (state space)	The space spanned by the state variables of a dynamical system.
Spatial resonance	Self-adjustment of spatial periodicity (wavelength) of a patterned state to an imposed external periodicity.
Saddle point	An unstable system state that has both stable and unstable manifolds, that is, directions of convergence and departure in phase space.
Saddle-node bifurcation	A collision and disappearance of two states of a dynamical system, as a control parameter traverses a threshold value.
Stable manifold	The set of points (initial conditions) in phase space that converge at long times to the system state.
Stable state	A system state that recovers from any sufficiently small perturbation or disturbance.
Unstable manifold	The set of points (initial conditions) in phase space that converge to the system state when time is run backwards.
Unstable state	A system state that evolves towards a different state when subjected to small perturbations.
Wavenumber	The spatial frequency of a periodic pattern, defined as the reciprocal of the wavelength.

The significance of studying the dynamics of self-organization lies in the ability to uncover their characteristic spatio-temporal modes. These modes, hereafter ‘self-organization (SO) modes’, represent ecological processes that direct ecosystems toward various stable states. Metaphorically, they are the road signs that point toward the possible destinations of self-organization. Mathematically, they are *growing eigenmodes* associated with *instabilities* (bifurcations) of ecosystem states [14], which dictate the growth directions of perturbations around unstable ecosystem states.

Here, we put forward the thesis that by analyzing specific ecological contexts of interest, exploiting methods of dynamical-systems and *pattern-formation theories* [14–18], concrete suggestions for human intervention can be made that meet the requirement of high ecological integrity under conditions of environmental variability, that is, keep the system at a high degree of self-organization, despite the intervention.

The simplest realization of this thesis is the much discussed topic of ecological thresholds [13, 19–21], where the implicit assumption is the existence of an unstable ecosystem state [22–24] that acts as a barrier, and thus as a threshold, for transitions between two alternative stable states. The simplicity in the notion of ecological thresholds makes it easily applicable, but at the same time it limits its usefulness. This univariate representation of the ecosystem focuses on whether a single process (representing a single SO mode), such as biomass removal, goes past a threshold, and thus limits management options to keeping the ecosystem within some safety bounds. However, the multivariate nature of most ecosystems and their spatial extent calls for generalizing that approach by considering the multidimensional *phase space* (state space) spanned by all relevant SO modes, and the *unstable states* it contains. These unstable states, usually neglected because no real-life ecosystem converges to them, strongly affect the flow in phase space; not only do they divide the phase space into distinct *basins of attraction*, and thereby determine the possible ecosystem trajectories that disturbances can induce, but this division may change as unstable states appear or disappear as a result of environmental changes. Viewing specific forms of human intervention as initial points in that phase space, informed choices of intervention, based on deep understanding of the phase-space structure, may avoid undesired outcomes.

Out of all contexts of ecological self-organization, dryland ecosystems stand out as excellent case studies for exploring high-integrity human intervention. Drylands are home to over a third of the world’s population, and a variety of research questions related to human intervention and the escalating concerns about desertification and biodiversity loss have been raised [5, 25]. Drylands also show striking spatial self-organization phenomena, which are well accounted for by dryland-vegetation models [26–32], and are readily accessible via remote sensing methods [33–35]. While dryland landscapes represent infinite-dimensional systems (because of their spatial extent), the actual dynamics may be governed by a small number of SO modes associated with a few instabilities of ecosystem states. Vegetation models indeed uncover two generic instabilities: bare soil losing stability to uniform vegetation, and uniform vegetation losing stability to periodic vegetation patterns [36]. Dryland-vegetation models therefore provide an excellent tool to study high-integrity human intervention problems that take into account more of the inherent dynamical complexity of actual ecosystems.

We focus in this paper on two contexts of human intervention in drylands: grazing management in drought-prone grasslands, and rehabilitation of degraded landscapes by water-harvesting methods. Through these two examples, we will show that the consideration of the inherent ecosystem dynamics provides essential information needed for maintaining high ecological integrity. We use model analysis to consider both cases, and show that the results remain valid even when strong environmental stochasticity is taken into account. We further analyze aerial images of an afforestation project to demonstrate actual dynamics driven by emerging SO modes. We conclude with a discussion of available empirical support for the proposed approach and the need for further empirical and theoretical studies.

## Results

### General approach

The question we address here is how to intervene in an ecosystem and maintain it near a self-organized functional state, despite environmental fluctuations and the additional stress that the intervention itself incurs. We first propose a general approach for high-integrity human intervention of this kind, and then demonstrate it with two examples, grazing management in drought-prone grasslands, and rehabilitation of degraded landscapes by water-harvesting methods.

The general approach consists of the following steps:

1. Identification of self-organized (SO) modes and the variables that quantify them, hereafter ‘SO variables’.
2. Uncovering the structure of the phase space spanned by the SO variables.
3. Exploration of high-integrity human intervention forms as initial phase-space points from which phase trajectories emanate toward functional ecosystem states.

Implementing these steps for a given ecological context may turn out to be too hard without a conceptual simplification of that context. In the two examples presented below, the context is dryland ecosystems, and the conceptual simplification involves the consideration of primary producers only, specifically plants, and a single limiting resource—water. This simplification necessarily misses significant aspects of the ecosystem’s complexity such as plant-soil feedbacks, species interactions and biodiversity, as well as aspects of the complex human impact on ecosystems [5, 6, 21, 37]. However, in drylands it is a reasonable simplification due to the dominance of plant-water interactions and the often existence of a single pattern-forming species [18].

Given these conditions, the analysis can rely on existing mathematical models that capture various pattern-forming feedbacks, and account for a wide variety of observed vegetation patterns [14, 36, 38]. More complex situations, e.g. where plant-soil feedbacks play important roles in addition to plant-water interactions, can be studied as well, but are not considered here [39]. The general approach may also be implemented in the absence of mathematical models, when detailed empirical data, including high-resolution remote-sensing images and rainfall patterns, are available. In this case it, is important to make sure that the scales of the empirical data and relevant phenomena match, i.e., that spatial and temporal scales are large enough to capture the spatial and temporal patterns of interest.

### Grazing management

The intervention goal in the grazing-management example we consider here is a provisioning ecosystem service—feeding livestock. High-integrity management means achieving this goal while maintaining the system in a viable vegetation state, despite the occurrence of occasional droughts.

The first step of high-integrity intervention is identifying the system’s SO modes. These are *growing eigenmodes* associated with instabilities of ecosystem states, which define the directions along which the system flows away from unstable states. If a reliable mathematical model of the particular ecological context of interest is available, then these instabilities and the associated SO modes can be identified using *linear stability analysis* [14].

**Fig 1:**
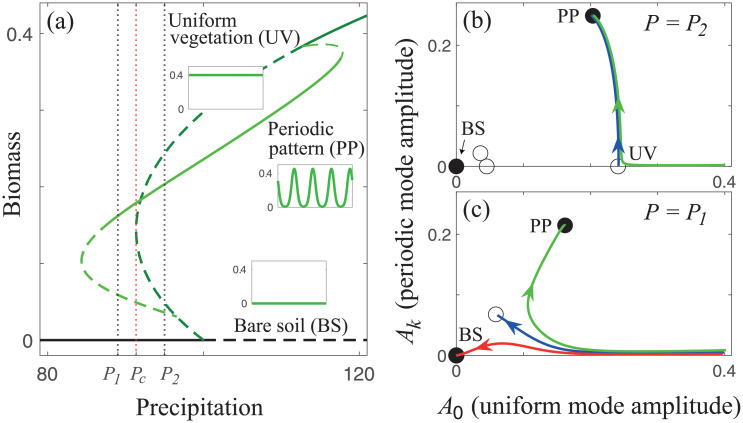
Managing grazing in grasslands. **(a)** A partial bifurcation diagram obtained from Eq (1), where solid (dashed) lines represent stable (unstable) states. The diagram shows three states: bare soil (black line, BS), uniform vegetation (dark green line, UV), and periodic vegetation pattern (light green line, PP). The insets show spatial biomass distributions of these three states. The uniform vegetation state disappears in a saddle-node bifurcation (fold bifurcation) at precipitation *P*_*c*_. **(b,c)** Phase space spanned by the self-organized (SO) variables *A*_0_ and *A*_*k*_ at precipitation *P*_2_ > *P*_*c*_ and at *P*_1_ < *P*_*c*_, respectively. Solid (open) circles represent stable (unstable) states. They correspond to the intersection points of the black vertical dotted lines in panel (a) with the various solution branches. The lines in blue represent invariant manifolds (stable or unstable) associated with two *saddle points*. At *P* = *P*_2_, where unstable uniform vegetation states still exist (two open circles on the horizontal axis in panel b), phase trajectories that emanate from a nearly uniform-vegetation state converge to a periodic pattern (green line in panel b). By contrast, at *P* = *P*_1_, where uniform vegetation states no longer exist, phase trajectories collapse to bare soil (red line in panel c). However, introducing a small component of the periodic mode *A*_*k*_ to the initial uniform state, which may represent non-uniform grazing, results in convergence to periodic pattern (green line in panel c). Units: precipitation [mm/yr], biomass and SO variables *A*_0_ and *A*_*k*_ [kg/m^2^].

Here, we use a relatively simple model of dryland vegetation [18, 31], given by Eq (1) in the Materials and methods section. This model, and similar ones, have been used over the past decade to predict and understand dryland patterns with notable success [34, 35, 40, 41]. These models are particularly relevant to understand periodic patterns that occur when the terrain is relatively homogeneous, and here we focus on such ecosystems. Fig 1a presents a *bifurcation diagram* that shows the existence and stability ranges of various ecosystem states in one spatial dimension along the rainfall gradient.

A uniform vegetation state is stable at high precipitation *P*, while for low *P* a bare soil state with no vegetation is stable. For intermediate *P* a periodic vegetation pattern state is stable, creating bistability regions where both the patterned state and either the uniform-vegetation state (high *P*) or the bare-soil state (low *P*) are stable. Mathematically, the two vegetated states in this system are the result of instabilities: A *uniform instability* of the bare-soil state, where the growth of a spatially uniform SO mode leads to a uniform vegetation state, and a *nonuniform (Turing) instability* of the uniform-vegetation state, which involves the growth of a spatially periodic SO mode to form a periodic pattern. The SO variables that quantify these modes can be chosen to be the time-dependent modes’ amplitudes, denoted here as the *A*_0_ for the uniform mode and *A*_*k*_ for the periodic mode, which can be deduced from spectral analysis.

Once the SO variables have been identified we can proceed to the second step—studying the structure of the phase space they span. Fig 1b and 1c show the phase-space spanned by *A*_0_(*t*) and *A*_*k*_(*t*) for two precipitation values, *P*_1_ and *P*_2_, that represent droughts of different strengths occurring in a system that had originally been in a stable uniform vegetation state. The main phase-space elements shown are: (i) uniform states and periodic vegetation states denoted by circles, where solid (open) circles correspond to stable (unstable) states, (ii) *Stable and unstable manifolds* of selected unstable states (blue lines), consisting of sets of points in phase space that flow toward unstable states (stable manifolds) or away from them (unstable manifolds), and (iii) phase trajectories emanating from phase-space points that represent initial states—the original uniform vegetation state before the occurrence of droughts, slightly perturbed (green and red lines). The phase trajectories and the manifolds were calculated by integrating numerically the model equations, as described in the Materials and methods section, starting with initial conditions of high uniform vegetation and extracting the SO variables *A*_0_ and *A*_*k*_ from spatial *spectral densities* [42, 43]. Note that circles denoting uniform states lie on the *A*_0_ axis as they do not have a periodic component, while circles denoting periodic patterns do not lie on the *A*_*k*_ axis as they have a uniform component that rules out negative biomass values.

We are in a position now to proceed to the third step and explore forms of high-integrity grazing management. The precipitation downshift to *P*_2_ represents a mild long-lasting drought that renders uniform vegetation unstable and results in a transition to a periodic pattern, as the green phase trajectory in Fig 1b shows. The precipitation downshift to *P*_1_ represents a stronger drought that results in a collapse to bare soil, despite the existence of a stable pattern state, as the red phase trajectory in Fig 1c shows. What makes the difference between these two dramatically different responses is the disappearance of the pair of unstable uniform-vegetation solutions in a *saddle-node bifurcation* at *P*_*c*_ (see Fig 1a). Above *P*_*c*_ the unstable manifold of the unstable uniform-vegetation solution (blue line in Fig 1b) acts as a barrier to the flow in phase space that starts from initial conditions of fairly uniform vegetation, preventing the approach of phase trajectories to the bare soil state. As the phase spaces show (and cannot be gleaned in the bifurcation diagram alone), below *P*_*c*_ this barrier no longer exists, and collapse to bare soil becomes possible.

The phase space information contained in Fig 1c leads to a significant insight about grazing management. It suggests that managing grazing in a non-uniform manner, so as to create a component along the periodic mode, *A*_*k*_(*t*), will increase ecosystem resilience to droughts. This is because of another manifold that acts as a barrier to phase trajectories (blue line in Fig 1c); points below this manifold (*A*_*k*_ too small) initiate phase trajectories that converge to the bare soil state, while points above this manifold (*A*_*k*_ large enough) initiate trajectories that converge to the periodic-pattern state. The latter correspond to nonuniform perturbations of uniform vegetation that can be interpreted as nonuniform grazing. In this view, grazing is introduced into the model simulations through initial biomass distributions or pulse perturbations. That view can be justified in cases of short grazing events relative to the time scale of vegetation growth.

**Fig 2:**
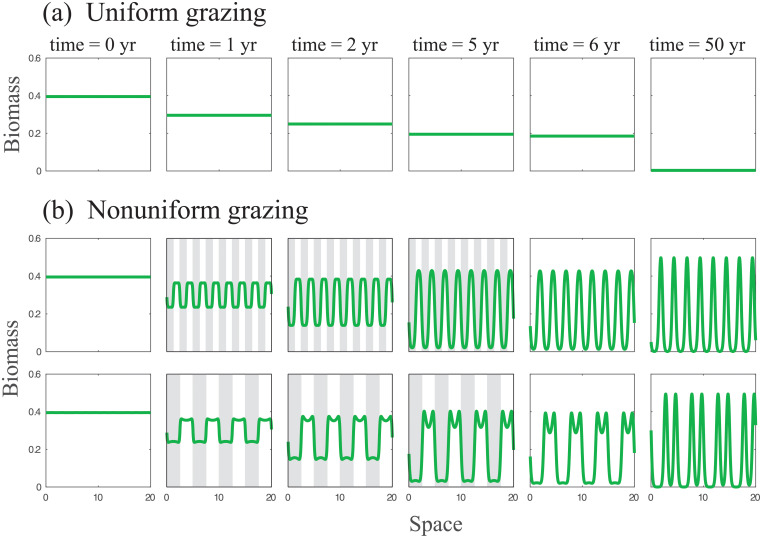
Response of the ecosystem to a drought under different grazing practices. Vegetation undergoes grazing in either uniform (a) or non-uniform (b) ways, modeled by modifying the biomass-decay parameter *M* in Eq (1). Snapshots taken over time (left to right) show the vegetation profiles, for a system undergoing a strong drought at *t* = 0, from precipitation *P* = 115 to *P* = 90[*mm*/*yr*] (similarly to red and green lines in Fig 1c). Uniform grazing is enacted by keeping a constant *M* = 2, while non-uniform grazing is modeled by having the system’s domain split into 16 (8) segments in the top (bottom) rows of panel b, where only odd segments undergo grazing for the first five years of a drought (marked by gray shading in the panels). During non-uniform grazing, values of *M* for grazed segments and non-grazed segments are *M* = 2.2 and *M* = 1.8, respectively.

Continual nonuniform grazing, where grazing proceeds continuously in time and nonuniformly in space, can be studied as press perturbations by modulating the biomass-decay parameter *M* (see Eq 1 and Table 2) in space. Fig 2 shows an example of model simulations with spatially periodic modulations of the biomass-decay parameter *M* applied to a limited period of time. The management scenario we envision here consists of switching from uniform grazing to nonuniform grazing once a developing drought is monitored, until a periodic vegetation pattern—a stable state at the reduced precipitation rate—develops. Practically, the grazing field is divided into equal lots and grazers are restricted to any alternate lot. Once the patterned state is established the management is switched back to uniform grazing, where grazers are no longer restricted in space. As Fig 2 shows, nonuniform grazing of this kind results in increased-resilience to droughts as compared with uniform grazing, and is not sensitive to the spatial periodicity of the nonuniform grazing.

**Table 2:** Model parameters.

Parameter name	Symbol	Units	Model A	Model B Figs 3 & 5	Model B Fig 6
Carrying capacity	*K*	*kg*/*m*^2^	1	5	10
Biomass decay rate (mortality and grazing)	*M*	1/*yr*	2	3	5
Evaporation rate	*N*	1/*yr*	5	10	7
Growth rate	Λ	(*kg*/*m*^2^)^−1^ *yr*^−1^	0.1	0.1	0.15
Water consumption rate	Γ	(*kg*/*m*^2^)^−1^ *yr*^−1^	4	10	5
Root-to-shoot ratio	*E*	(*kg*/*m*^2^)^−1^	1.5	-	-
Shading coefficient	*R*	−	0.1	-	-
Max infiltration	*A*	1/*yr*	-	100	50
Infiltration reference	*Q*	*kg*/*m*^2^	-	0.25	1
Infiltration contrast	*f*	−	-	0.1 − 0.2	0.1 − 0.2
Biomass spread	*D*_*B*_	*m*^2^/*yr*	0.01	0.02	0.4
Soil-Water diffusion	*D*_*W*_	*m*^2^/*yr*	10	2	0.1
Water overland spread	*D*_*H*_	(*m*^2^/*yr*)/(*kg*/*m*^2^)	-	2	5
Precipitation	*P*	(*kg*/*m*^2^ ⋅ *yr*^−1^)	80 − 120	200 − 360	150 − 250

The surprising conclusion we draw from this analysis is that managing grazing non-uniformly is a high-integrity form of human intervention that can result not only in achieving an ecosystem service but also in increasing the resilience to droughts, rather than decreasing it due to the additional stress that uniform grazing imposes.

### Rehabilitation of degraded landscapes

A common approach of rehabilitating degraded vegetation is water harvesting by spatially periodic ground modulations that intercept overland water flow and along which vegetation is planted [44]. Most often, the ground modulations consist of micro-catchments, such as parallel contour bunds or furrows, but milder intervention forms, such as soil-crust removal, can also be envisaged. In the following we study vegetation rehabilitation by a stripe-like configuration of ground modulations using another variant of the Gilad et al. model given by Eq (3) in the Materials and methods section. We modulate the infiltration rate, as in Eq (5), to mimic stripes of removed soil crust that form a periodic configuration in the *x* direction with a *wavenumber* *k*_*f*_ or wavelength *L*_*f*_ = 2*π*/*k*_*f*_. This is a *spatial resonance* problem [45, 46] whereby a system that tends to self-organize in a periodic pattern with a wavenumber *k*_0_ (wavelength *L*_0_) is subjected to an external periodic force of a different wavenumber *k*_*f*_.

The natural wavelength *L*_0_ is determined by various biotic and abiotic factors, such as the lateral root extension and the rates of precipitation and infiltration, and when it differs from the forcing wavelength *L*_*f*_ it stands in conflict with the favorable growth conditions that the latter forms. Since *L*_0_ changes with environmental conditions and cannot be known in highly variable environments, a major question of resilience arises: what plantation patterns keep productive system states most resilient to environmental changes? Should the plantation pattern follow the periodic configuration of ground modulations, i.e. vegetation bands along each stripe of removed crust, or should other plantation patterns, that do not fully overlap the periodic ground modulations, be used instead?

**Fig 3:**
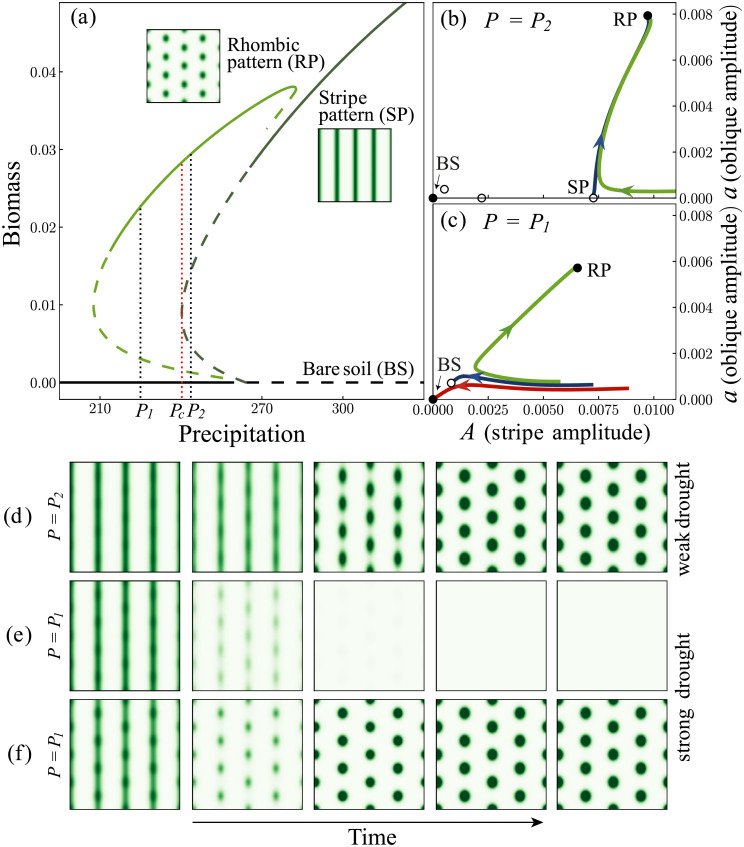
Vegetation rehabilitation using periodic ground modulations. **(a)** A partial bifurcation diagram obtained from Eq (3), showing bare soil state (black line, BS), a stripe pattern (dark green line, SP) and a rhombic pattern (light green line, RP). Solid (dashed) lines represent stable (unstable) states. The insets show examples of two-dimensional spatial biomass distributions of the two patterned states. The stripe pattern disappears in a saddle-node bifurcation at precipitation *P*_*c*_. **(b,c)** Phase space spanned by the self-organized (SO) variables *A* and *a* above the saddle-node bifurcation (*P* = *P*_2_ > *P*_*c*_) and below it (*P* = *P*_1_ < *P*_*c*_), respectively. Solid (open) circles represent stable (unstable) states. They correspond to the intersection points of the black vertical dotted lines in panel (a) with the various solution branches. The lines in blue represent stable and unstable manifolds. At *P* = *P*_2_, where the unstable stripe pattern still exist, phase trajectories that emanate from a nearly stripe-pattern state converge to the rhombic pattern (green line in panel b). In contrast, at *P* = *P*_1_, where the stripe-pattern state no longer exists, phase trajectories collapse to bare soil (red line in panel c). However adding small components of the oblique modes to an initial stripe pattern places the system above the stable manifold of the unstable rhombic pattern and results in convergence to the rhombic pattern (green line in panel c).**(d-f)** Snapshots of a 13 × 13 [m] domain, taken over time (left to right) showing response of ecosystem to a drought, corresponding to green line in panel b, and red and green lines in panel c, respectively.

In order to study this question we first identify the relevant SO modes. The bifurcation diagram in Fig 3a reveals two basic instabilities: an instability of the bare-soil state to a periodic stripe pattern as the precipitation parameter is increased past a threshold value, and an instability of the periodic stripe pattern to a spot-like rhombic (stretched hexagonal) pattern [47] as the precipitation parameter is decreased below another threshold value. Fig A in S1 Appendix illustrates the development in time of the two instabilities. Notice that the rhombic pattern persists as a stable state at precipitation values significantly lower than that of the stripe pattern.

**Fig 4:**
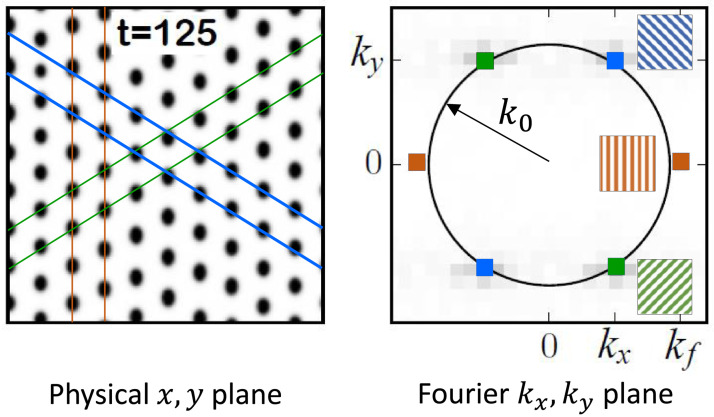
Self-organized (SO) modes associated with rhombic pattern. A nearly asymptotic rhombic pattern in the (*x*, *y*) plane (left), resulting from an instability of a stripe pattern, and the corresponding Fourier plane (*k*_*x*_, *k*_*y*_) showing the three SO modes that comprise it (right). See Fig A in S1 Appendix for earlier snapshots. These modes include the original stripe mode with wave-vectors ±(*k*_*f*_, 0) (red squares in right panel), representing periodicity in the *x* direction as the inset with vertical red stripes in the right panel show. The signature of this mode in the rhombic pattern (left panel) is illustrated by the red lines. The two additional SO modes are a pair of oblique modes with wave-vectors ±(*k*_*f*_/2, *k*_*y*_) and ±(*k*_*f*_/2, −*k*_*y*_) (blue and green squares, respectively, in right panel), which represent slanted stripe patterns as the insets in blue and green in the right panel show. The wave-vector component *k*_*y*_ is determined such that the corresponding wave-vector sits on a circle of radius *k*_0_—the wavenumber (periodicity) that the natural pattern (in the absence of ground modulations) tends to form. The signatures of the oblique modes in the rhombic pattern (left panel) are illustrated by the slanted blue and green lines.

The instability of the bare-soil state involves the growth of a stripe SO mode of the form *A*(*t*) cos(*k*_*f*_*x*) (up to an arbitrary constant phase), where *A*(*t*) is the time-dependent mode’s amplitude. The growth of this periodic mode, as precipitation is increased, represents the expected establishment of vegetation along the stripes of removed soil crusts, where the infiltration rate and thus the soil-water content are higher. The instability of the stripe pattern involves the growth of two additional SO modes of the form *a*(*t*) cos(*k*_*x*_*x* ± *k*_*y*_*y*), where *a*(*t*) is the shared amplitude of the pair of modes, *k*_*x*_ = *k*_*f*_/2 and *k*_*y*_ is such that the total wavenumber *k* is equal to the natural wavenumber *k*_0_ (see Fig 4) [45]. The appearance of these modes can be deduced using a *spectral-density analysis* of the rhombic pattern they lead to. Fig 4 shows a rhombic pattern (left panel) and the modes of highest amplitudes (colored squares) in the (*k*_*x*_, *k*_*y*_) plane (right panel). The original stripe mode is represented by the red squares, whereas the two additional SO modes are represented by the blue and green squares. We call the latter “oblique modes” since each of them represents a slanted periodic stripe pattern, as the insets in Fig 4 show. The signature of the stripe mode in the rhombic pattern appears as a periodicity in the *x* direction (red lines), while the signatures of the obliques modes appear as periodicities in slanted directions (blue and green lines). The growth of the oblique SO modes from a stripe pattern, as precipitation is decreased, reflects vegetation mortality to form a spot (rhombic) pattern. Each spot benefits from a larger area of surrounding bare soil, and thus from an additional source of water through source-sink relations [48]. The SO variables that quantify the SO modes are chosen to be their time-dependent amplitudes, *A*(*t*) of the stripe mode, and *a*(*t*) of the symmetric pair of oblique modes.

The existence of two functional ecosystem states, stripe and rhombic patterns, opens up two options for restoring degraded vegetation, as Fig B in S1 Appendix illustrates: continuous plantation along the modulation stripes to initiate the stripe mode and its convergence to a stripe pattern, or fragmental plantation to initiate the two oblique modes and their convergence to a rhombic pattern. We argue that although both pattern states (and the plantation pattern they represent) are functional, they differ in their resilience to droughts, and thus in their long-term ecological integrity. Crucial to our argument are the roles that unstable states play in dividing the phase space into distinct domains through their stable and unstable manifolds. While in the grazing-management example considered in the previous section it was the unstable uniform-vegetation state that prevented collapse to bare soil, here the unstable stripe pattern plays a similar role.

We consider first the response of an ecosystem that has been restored in a stripe pattern to a moderate precipitation downshift to a range where the stripe pattern is unstable but still exists (*P* = *P*_2_ in Fig 3a). As the green trajectory in Fig 3b and the snapshots in Fig 3d show, the downshift results in a smooth transition to a rhombic pattern. However, stronger downshifts, to precipitation values (*P* = *P*_1_ in Fig 3a) below the saddle-node bifurcation at which the unstable stripe pattern disappears, can result in a collapse to bare soil, as the red trajectory in Fig 3c and the snapshots in Fig 3e demonstrate. This is because the stripe pattern and its unstable manifold no longer exist to direct phase trajectories towards the stable rhombic pattern. The initial stripe pattern lies within the basin of attraction of the bare-soil state and the phase trajectory converges to this state. This behavior should be contrasted with that of an ecosystem that has been restored in a rhombic pattern. In this case, a strong precipitation downshift to *P* = *P*_1_ will result in a convergence to a rhombic pattern, while a precipitation upshift to a range where rhombic patterns are unstable or do not exist, representing rainy years after drought, will result in a smooth transition towards a stable stripe pattern.

Importantly, the nearly stripe pattern obtained in this process might not suffer from the poor resilience of a restored stripe pattern, because it is likely to contain vestiges of the oblique modes. These oblique modes can place the system above the dividing manifold, in the basin of attraction of the stable rhombic pattern (see the green trajectory in Fig 3c and the snapshots in Fig 3f). In conclusion, our model analysis suggests that rehabilitation of drought-prone ecosystems in rhombic patterns, rather than in stripe patterns, should result in longer-term productivity and therefore represents rehabilitation of higher-integrity.

### Robustness to environmental stochasticity

**Fig 5:**
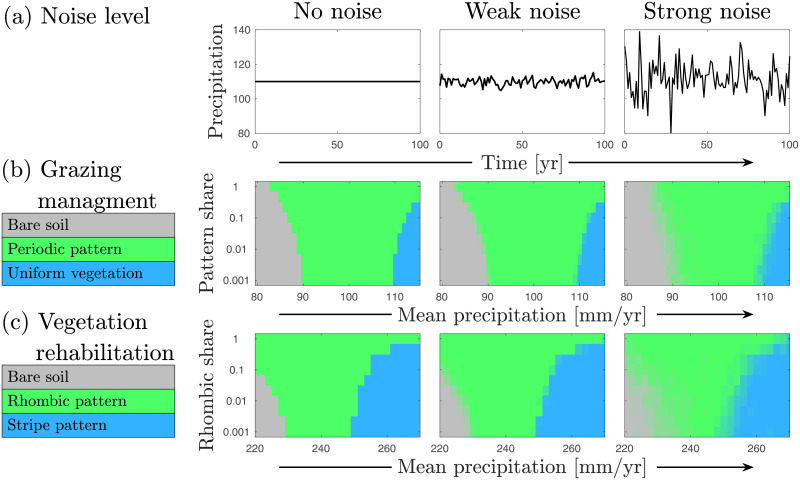
Responses to precipitation downshifts under stochastic precipitation and different initial conditions of mixed vegetation states. Left, middle and right columns correspond to negligible, weak and strong precipitation fluctuations, respectively. **(a)** Demonstration of noise level. **(b)** Asymptotic states (see color legend) for the grazing management system, where initial conditions consist of increasing portions of periodic pattern in uniform vegetation (pattern share). **(c)** Asymptotic states for the vegetation rehabilitation system, where initial conditions consist of increasing portions of rhombic pattern in stripe pattern (rhombic share). Each pixel in the parameter plane (mean precipitation—share) shows the asymptotic state obtained from averaging over 20 simulations with a unique randomization of temporal noise from a Gamma distribution per simulation, where the initial conditions correspond to mixtures of states calculated at *P* = 115[mm/yr] (*P* = 260[mm/yr]) for middle (bottom) row. Note that this vertical axis is logarithmic.

The results described so far were obtained by solving Eqs (1) and (2) with constant precipitation rates, where precipitation downshifts were captured by initial states that were calculated at higher constant precipitation values. To what extent do these results remain valid under more general conditions of environmental stochasticity, such as due to fluctuating rainfall? To answer this question we studied the model equations using a precipitation rate that changes annually, with random values taken from a Gamma distribution [49] We compared three cases: no noise, weak noise and strong noise, realizations of which are shown in Fig 5a. For each case we considered initial conditions involving superpositions of two states as follows: increasing portions of a periodic-pattern component in a superposition with uniform vegetation (“pattern share”) for the grazing management problem (vertical axis in Fig 5b), and increasing portions of a rhombic-pattern component in a superposition with a stripe pattern (“rhombic share”) for the rehabilitation problem (vertical axis in Fig 5c). The basic states in these mixed initial conditions, i.e. uniform vegetation in the grazing management problem and stripe pattern in the rehabilitation problem, were calculated at high precipitation and the outcomes of precipitation downshifts to the prescribed mean precipitation values on the horizontal axes in Fig 5b and 5c were studied under the three aforementioned precipitation cases. The outcomes are of three types: no change in the basic state (blue domains), shift to an alternative functional state (green domains), and collapse to bare soil (grey domains). As the figure indicates, the overall response remains the same, irrespective of the noise level. Higher proportions of the alternative state in the initial conditions mean that the system is less liable to collapse, as implied by the shrinking grey domains.

### Empirical observations of SO modes

The SO modes are central to the proposed approach for high-integrity human intervention, as the model studies described so far demonstrate. But, are there empirical indications for their existence, and for the pattern change that their growth induces? Observations of SO modes, indicating the development of periodic vegetation patterns from uniform vegetation following a prolonged drought, have been reported in studies of vegetation patterning over a period of 40 years in southern Niger [50]. These results support the construction of a phase space spanned by a uniform SO mode and a periodic SO mode, as was done in the grazing management example. Here we bring a new evidence for the emergence of oblique SO modes in afforestation projects in the northern Negev, Israel [14, 51]. These projects involved the engineering of furrows along topographical contours, to harvest runoff water, and the plantation of trees along them to make use of the increased soil-water content.

**Fig 6:**
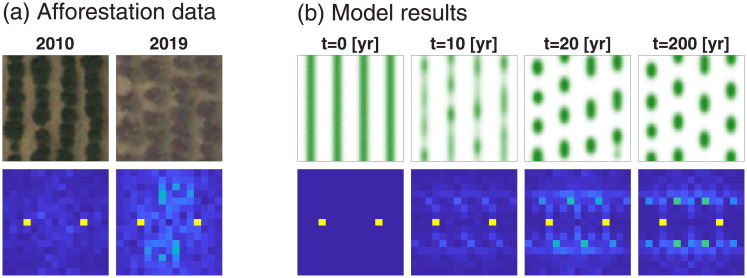
Emergence of oblique self-organized (SO) modes in afforestation projects. (a) A region of size 30x30 [m] containing four stripes of planted trees along bunds, taken from aerial images of the northern Negev region (Coordinates: 31.295N, 34.815E) in 2010 and 2019. (b) Model results of a comparable system, consisting of four initial vegetation stripes that has been subjected to a precipitation downshift from *P* = 205 to *P* = 180 [mm/yr] at *t* = 0, and simulated to *t* = 200[yr] (see full details in Materials and methods section and S1 Appendix). Note that similarly to Fig 3, but with different values of *P*, at high precipitation (*P* = 205) both stripe and rhombic patterns are stable, but at low precipitation (*P* = 180) stripe patterns are no longer stable. Top row shows spatial images, while bottom row shows spectral densities obtained from spectral (FFT) analysis, which demonstrates the periodicity of vegetation along different directions. The empirical spectral density in 2010 (a) shows the dominance of a stripe SO mode (yellow dots on *x* axis), representing the original planted pattern, while that in 2019 shows, in addition, the development of oblique modes (light-blue dots off the *x* axis), which represent vegetation mortality to form a spot-like pattern. The model simulations in (b) show a similar trend. During the transient dynamics towards a rhombic pattern the emerging pair of oblique modes are not symmetric (compare with Fig 4) both in the empirical data and the model simulations. However, simulations to longer times (*t* = 200[yr]) indicate the eventual emergence of a symmetric pair of oblique modes.

Using aerial images taken in 2010, we identified 100 small regions of 4 parallel bunds each, where the planted trees grew to form a stripe pattern, and compared those regions in 2010 to their state in 2019. Fig 6 presents an analysis of a sample region and, for qualitative comparison, also model results that show the response to a press perturbation in the form of a long drought. In this model analysis we used a new parameter set (Table 2), that better fits the spatial and temporal scales of the ecosystem, as well its precipitation regime. The data of 2019 (panel a) show a fragmentation of the stripe pattern into a spot-like pattern, which translates in the spectral-density analysis to the appearance of oblique *Fourier modes*, not aligning along the horizontal *k*_*x*_ axis. As the model results in panel (b) (*t* = 10 yr) suggest, this is a transient behavior that slowly approaches a rhombic pattern. This behavior should be contrasted with a response that does not involve the growth of oblique modes and results in a uniform biomass decline along the stripes, keeping the stripe morphology unchanged. That response does not occur, apparently because of the scale-dependent feedback between biomass and water that is typical of dryland vegetation and results in vegetation patterning [48, 52]. Overall, 64 out of the 100 regions surveyed had signatures of oblique modes, implying that a transition to a rhombic pattern was indeed taking place across the planted forest. An additional 9 regions showed significant degradation with more than half the trees dying, possibly indicating a collapse to bare soil, as can occur, according to our theoretical analysis, due to lower rainfall rates or less perturbed initial stripes. Of the remaining 27 regions, 9 did not show a significant change, and 18 were difficult to classify—both responses possibly occurring due to stronger heterogeneities (e.g. occurrence of rocks in soil) in those regions, that would affect the response of vegetation to drought.

We attribute the change in tree pattern to drought pulses that occurred in 2008–2009 and 2017–2018, and involved rainfall drops of 50–70 mm/yr below the mean annual rainfall of 179 mm/yr during the period 2004–2019, combined with a press-like decline in the mean annual rainfall from a higher value of 208 mm/yr during the years 1980–2003 (see Fig D in S1 Appendix). Drought pulses can result in several responses, including a reduction in leaf area and crown “dieback”, where only a portion of a tree’s canopy dies [53], an increase in individual tree mortality [54], and broad-scale die-off events [55]. In the present case of a runoff harvesting system of planted tress, the pattern change is likely a result of dieback and individual tree mortality that were amplified by the cumulative stress that was built up following two successive drought pulses [56]. An amplification effect of this kind has been reported in long term studies of woody plants in the same study area (Park Shaked LTER) [57]. We refer the reader to the Materials and methods section and to S1 Appendix for more details about this analysis.

## Discussion

We presented here a general theoretical approach for high-integrity human intervention in ecosystems, using dryland vegetation as a case study. The novelty in this approach lies in the identification of the few inherent self-organization (SO) modes that drive the system from one state to another, the derivation of their dynamics, and the utilization of these modes to study human intervention forms that result in fast convergence to functional states, despite the additional stresses that these interventions often exert. This approach has been motivated by an earlier model study of rehabilitation by water harvesting [46], where a simple toy model of pattern formation—the Swift-Hohenberg equation [14]—was used to derive the reduced dynamics of the SO modes. Here, we use a fairly elaborate water-vegetation model (Eq 3) to derive these dynamics, and demonstrate the generality of the approach by considering an additional example—grazing management in drought-prone grasslands. The analysis of the grazing example has led to a surprising novel result, namely, that nonuniform management of grazing increases the resilience to droughts, as compared with uniform grazing that exerts an additional stress on already vulnerable ecosystems.

In implementing this approach, special attention has to be paid to unstable states and their stable and unstable manifolds, which affect the dynamics in phase space, and thereby, the asymptotic state that the system approaches. Thus, in the example of grazing management in drought-prone grasslands we have identified vulnerability to desertification, associated with the disappearance of an unstable uniform vegetation state. We further found that managing grazing non-uniformly can reduce the risk of desertification by creating a component along a periodic SO mode, which directs the ecosystem towards a functional patchy vegetation state. From an ecological point of view, the increased resilience to droughts, achieved with non-uniform grazing, can be understood as a consequence of the positive water-biomass feedback loop [48] that gives competitive advantage to ungrazed (higher-biomass) patches over their grazed (lower-biomass) neighbors in capturing the limiting water resource. This competitive advantage results in reduced competition and increased resilience to potential droughts.

A similar situation appears in the vegetation-rehabilitation problem, where the common practice of rehabilitation in stripes (Fig B in S1 Appendix) results in vulnerability to strong droughts, whereas fragmental rehabilitation (Fig B in S1 Appendix) that contains components of oblique modes, results in the more resilient rhombic patterns. Here too, the vulnerability of stripes to desertification is associated with the disappearance of an unstable state—the stripe-pattern state—and the creation of components along the oblique modes is a means to reduce the competition, this time along the ground modulations. The analysis of this example highlights the significance of distinguishing between the ground modulation pattern and the vegetation planting pattern; the two need not coincide in variable environments where state transitions are likely to occur.

The analyses of these two examples highlight a novel mechanism of abrupt regime shifts that is not associated with the commonly conceived tipping-point behavior. The mechanism involves two instabilities, a primary instability of a dysfunctional state to a functional state, as environmental conditions ameliorate, and a secondary instability of the functional state to another functional state as environmental conditions worsen. Abrupt shifts are then associated with the disappearance of the unstable functional state. This mechanism is expected to apply to any system that shares this bifurcation structure, and, in particular, to ecosystems that have developed other forms of ‘defense mechanisms’ that play similar roles to that of spatial patterning in the present case, in order to cope with environmental stresses and remain functional. It shares with the simpler tipping-point mechanism the abrupt, global nature of the transition, but differs in two respects: (a) the transition requires a significant downshift of the environmental parameter (e.g. down to *P* < *P*_2_ in Fig 1), (b) the transition to the dysfunctional state can occur well before the tipping point, in a parameter range where the system still has a stable functional state, e.g., the transition to bare soil that follows the precipitation downshift to *P*_1_ in Fig 1c where a functional periodic pattern still exists as a stable state.

The projection for increased likelihood of extreme events, such as severe droughts, makes the new mechanism particularly relevant in the current era of climate change. However, the existence of an alternative functional stable state paves the way for devising preventive measures that direct the ecosystem towards this state, following an extreme event, by creating components of the appropriate SO mode. Such interventions may often integrate with needs for provisioning ecosystem services. This appears to be the case with non-uniform grazing or clear-cutting that build up components of periodic SO modes and induce transitions, following severe droughts, to functional patchy-vegetation states rather than collapse to bare soil.

Empirical evidence in support of the ideas presented here is still very limited, possibly because of the lack of studies intended to test them. Evidence for the improved resilience of non-uniform grazing may come from studies of rotational grazing, a practice that has been reported to be more sustainable than continuous grazing [58]. In rotational grazing, grazers are moved periodically among paddocks rather than let to graze on a single plot for the entire grazing season, thereby producing spatial periodicity. Further model and empirical studies are needed in order to explore optimal practices in terms of paddock size, grazing time in a paddock, number of paddocks simultaneously grazed, etc.

While vegetation restoration by periodic ground modulations is a common practice, long-term studies of restored areas that focus on large-scale spatial patterning are still lacking. Yet, indications of spatial self-reorganization along human-made ground modulations do exist, as we demonstrate in our remote-sensing data analysis (see Fig 6). As we show, long-term monitoring of such areas using remote sensing data can be used to calculate spatial spectral densities and identify the SO modes that drive the dynamics. In our aerial image analysis, we could see in many of the areas the development of oblique-mode peaks in the course of time, similarly to the simulation shown in Fig 3d and 3f. Although it is by no means conclusive, this result provides empirical support for the emergence of the pair of oblique SO modes and for the transition from stripe to rhombic pattern, as the theoretical analysis predicts. The model prediction that rehabilitation in rhombic patterns is more resilient than rehabilitation in stripe patterns, unfortunately, cannot be tested as relevant data are not available. This prediction, however, can serve as an informed hypothesis to be test in dedicated rehabilitation experiments with long-term monitoring. We note that our model (Eq 3) describes flat terrains whereas the empirical data are taken from gently slopped terrains. Adding a gentle slope is not expected to change the general results, as has been shown in an analysis of a simpler pattern formation model [59]. Moreover, the fact that the general response we see in our aerial image analysis is similar to model prediction hints at a universal behavior of such human modified ecosystems, and demonstrates that understanding the pattern formation properties of simple models can help understand complex dynamics in real ecosystems.

We focused here on dryland vegetation as a case study, but the general approach should be applicable to many other ecological contexts showing instabilities of uniform and periodic states. Candidate systems of immediate interest are those for which mathematical models have already been suggested. Example of such contexts are vegetation patterns in wetlands [60], spatial patterns in seagrass meadows [61], patterns in benthic bacteria-nutrient systems [62, 63], tidal morphodynamics [64], patterns in young mussel beds [65, 66] and others. In the case of drylands, we considered two components—the biomass of a dominant plant species and water—and the associated human intervention, e.g., grazing and water harvesting, in the form of pulse or press perturbations. Because of the tendency of dryland ecosystems to self-organize in spatial patterns these systems are still high dimensional, and the reduction to low-dimensional dynamics [67], as shown in Figs 1b and 1c and 3b and 3c, has been obtained by focusing on SO modes associated with instabilities that have incurred in the system. That approach is likely to be applicable to other contexts involving pattern-forming species, such as those noted above.

However, the consideration of additional elements of ecosystem complexity may require further development of the approach. One example are diverse plant communities that self-organize in space as a result of plant-resource feedbacks or plant-soil feedbacks [37]. Here, a trait-based approach, where vegetation biomass depends not only on physical space but also on trait space [32], may prove useful. Another example is evolutionary dynamics that involve spatial self-organization. Here, applying theoretical frameworks, such as adaptive dynamics [68], to vegetation models that capture spatial instabilities can be useful.

The results reported here highlight the significance of incorporating concepts of dynamical-system and pattern-formation theories into studies of high-integrity human intervention. These concepts can readily be used when faithful mathematical models are available, but should remain useful also in the absence of mathematical models, when detailed empirical data, such as high-resolution airborne images, are available. Highly resolved spatial time series, for example, may be used to extract the dynamics of the few primary spatial Fourier modes—the SO modes—and construct the structure of the phase space they span, as was done in creating Figs 1 and 3 using model simulations. More general contexts of ecosystem dynamics, not necessarily involving spatial patterning, call for the development of additional methods of dimension reduction, such as principal component analysis and others [69], for identifying SO modes and tracking their dynamics.

Combining mathematical analysis with empirical observations to uncover the phase-space dynamics of self-organization modes, using advanced methods of dimension reduction, can pave the way for novel high-integrity human-intervention forms and new practices of sustainable ecosystem management.

## Materials and methods

### Mathematical models

A general model platform for dryland vegetation [32] is applied to two different contexts, thereby producing two model variants: pattern-forming grassland ecosystem (Model A), and vegetation restoration by water harvesting (Model B). The application of the general model platform to the two specific contexts results in model simplifications, which facilitate model analysis but do not involve loss of essential information. The general platform consists of PDEs for the areal densities of above-ground biomass of all species (or functional groups) *B*_*i*_(*r*, *t*)(*i* = 1, …, *N*), soil water *W*(*r*, *t*), and surface water *H*(*r*, *t*), all in units of [kg/m^2^], where *r* = (*x*, *y*) [m] represents the spatial coordinates in the plane, and *t* [yr] represents time. We note that PDE modeling can be justified even for small plant populations, as is often the case in drylands [70]. In all contexts we assume flat horizontal terrains and plant species with laterally confined root systems for which the integral terms in the general platform, which describe nonlocal water uptake by laterally extended roots, can be calculated and transformed into algebraic forms [32]. Under these conditions, vegetation-water interactions, as well as water flow, are limited to small spatial scales, of the order of a few meters for the parameters used in our models [71, 72].

### Model A—Grassland ecosystem

In this context we assume a single grass species and a sandy soil for which the infiltration rate of surface water into the soil is high both in bare and vegetated areas. As a consequence, overland water flow is insignificant, and the equation for surface water can be eliminated. The general platform then reduces to the following simplified system of PDEs:

$$\begin{array}{cc} \partial_{t}B=G_{B}B(1-\frac{B}{K})-MB+D_{B}\nabla^{2}B, & \left(1\text{a}\right)\\ \partial_{t}W=P-LW-G_{W}W+D_{W}\nabla^{2}W, & \left(1\text{b}\right) \end{array}$$

where,

$$\begin{array}{c} G_{B}=\Lambda W{\left(1+EB\right)}^{2},\;G_{W}=\Gamma B{\left(1+EB\right)}^{2},\;L=\frac{N}{1+\frac{RB}{K}} \end{array}$$ (2)

are biomass growth rate, water-uptake rate, and soil-water evaporation rate, respectively. Explanations about all model parameters, and their values, appear in Table 2. Parameter values are based on [73] and [34].

### Model B—Vegetation restoration

Here we still assume a single plant species, woody or herbaceous, but consider a crusted soil with low infiltration rate of surface water, except at vegetation patches. The surface-water equation cannot be eliminated in this case and the model equations read

$$\begin{array}{cc} \partial_{t}B=G_{B}B(1-\frac{B}{K})-MB+D_{B}\nabla^{2}B, & \left(3\text{a}\right)\\ \partial_{t}W=IH-LW-G_{W}W+D_{W}\nabla^{2}W, & \left(3\text{b}\right)\\ \partial_{t}H=P-IH+D_{H}\nabla^{2}(H^{2}), & \left(3\text{c}\right) \end{array}$$

where *G*_*B*_, *G*_*W*_ and *L* are given by (2), and

$$\begin{array}{c} I=A\frac{B+Qf}{B+Q} \end{array}$$ (4)

is the surface-water infiltration rate that captures the different infiltration rates in bare-soil (low) and in vegetated soil (high) for *f* ≪ 1. Stripe-like ground modulations by removed soil crust are modeled by modulating the infiltration contrast, *f*:

$$\begin{array}{c} f=f_{0}\{1+\frac{\gamma_{f}}{2}[\text{cos}(k_{f}x)+1]\} \end{array}$$ (5)

with *f*_0_ the baseline infiltration contrast, *γ*_*f*_ the modulation strength, and *k*_*f*_ (2*π*/*k*_*f*_) the spatial wavenumber (wavelength) of the modulation. Explanations about values for all model parameters appear in Table 2. Parameter values for Figs 3 and 5 are based on dimensionalization of parameter values from a previous study [46]. Parameter values for Fig 6 were taken to represent a dryland ecosystem of woody vegetation, similar to the system for which aerial images were analyzed. These values were based on [73] and [34].

### Numerical analysis

The spatial distributions of the biomass density (Figs 1, 2, 3 and 6) and the phase-space trajectories (Figs 1 and 3) shown are the results of integrating in time the equations of the different models. This integration was done numerically using the Euler method for advancing time, while spatial derivatives were resolved using a finite difference scheme (a five-point stencil for the Laplacian in the two-dimensional system). Typical grid sizes used were 100 by 100 points for the two-dimensional system and 500 points for the one-dimensional system.

Each *phase-space trajectory* was simulated once, starting with an initial condition around the stable state at high precipitation (uniform vegetation at *P* = 115[*mm*/*yr*] for Fig 1, a stripe pattern at *P* ≈ 300 [mm/yr] for Fig 3), and integrating in time with the relevant precipitation value (*P*_1_, *P*_2_). Approximations to stable and unstable manifolds of a saddle point were found by starting with similar initial conditions to those of the phase-space trajectories, except that many different trajectories were scanned as part of a binary search for trajectories that spend increasingly long times near the saddle points, diverging, eventually to one side of the saddle or to the other side.

The SO variables shown in the phase planes (*A*_0_ and *A*_*k*_ in Fig 1, *A* and *a* in Fig 3) were found using spatial spectral densities [74, 75]. These spectral densities were calculated by taking the absolute value of a discrete Fourier transform (in one dimension in Fig 1, in two dimensions in Fig 3). More specifically, Numpy’s “ifft” function was used (FFT module), in order to ensure a correct normalization, independent of grid size. The SO modes where identified with the primary Fourier modes, disregarding harmonics. The SO variables were then associated with the absolute value of the Fourier modes.

The bifurcation diagram of Fig 1 was computed using the *numerical continuation* software AUTO [76]. The bifurcation diagram of Fig 3 was computed using the numerical continuation software pde2path [77]. Stability properties shown in these diagrams were calculated by extracting solutions and performing numerical linear stability analysis, and then validating these results using time integration. The parameter planes (mean precipitation—share) in Fig 5 were calculated by running 20 simulations per pixel shown in the panels (36x11 and 46x11 pixels per panel, for middle and bottom rows, respectively). In each simulation, a random time series for precipitation was generated (uncorrelated to time series of other simulations) with 100 values representing precipitation for each year. The values were taken from a Gamma distribution [49, 78] of average value *p*_0_ and standard deviation *σ* = *p*_0_ *σ*_0_, where *σ*_0_ is 0, 0.02, 0.1 for the left, middle and right columns, respectively. Examples of time series are shown in the top panels of Fig 5. Gamma distribution has been shown to be appropriate for representing the stochasticity associated with precipitation in drylands [78], and we also show in S1 Appendix that our results do not depend on the specific distribution, by using a Gaussian distribution, giving us the same qualitative results (Fig C in S1 Appendix).

For each simulation, an initial state is constructed from a mixture of two states of high precipitation. For the middle row these are the uniform vegetation and periodic pattern states (8 peaks in domain of 20[m]), taken at *P* = 115. For the bottom row these are the rhombic pattern and stripes pattern states (similarly to Fig 2), taken at *P* = 260. The mixture is determined by the position along the vertical axis of the panel, where 0.01 means a mixture of 1% of the periodic pattern (rhombic pattern) state and 99% of the uniform vegetation (stripe pattern) state, for the middle (bottom) row. This initial state is then integrated in time for 100 years using the time series as explained above, and the resulting pattern at the end of this time is evaluated using an automatic algorithm, based on biomass thresholds (for differentiation between patterns and uniform states) and detection of patch number (for differentiation between different types of patterns), with the color of the pixel chosen accordingly (see color legend in the left side of Fig 5).

Specifically, for the one-dimensional system, vegetated states had average biomass between 0.1 and 0.45, and almost all patterned states had variation of biomass between 0.4 and 0.6. Thus a biomass threshold of 0.05 was used for this system (average biomass smaller than threshold is bare soil, variation in biomass larger than threshold is patterned state, everything else is uniform vegetation). For the two-dimensional system, vegetated states had average biomass between 0.02 and 0.16, so that states with less than a threshold of 0.01 was deemed as bare soil. Stripe pattered states have multiple (4 in our case) regions of bare soil that are not connected, while all rhombic patterned have only 1 since the breakup of the pattern connected the bare soil regions into one large one. Thus patterns with more than one bare region were deemed as rhombic patterns, and other regions as stripe patterns.

### Analysis of remote sensing data

We chose for the case study an afforestation project in the northern Negev, Israel (coordinates: 31.295N, 34.815E). Aerial images were taken from the website of the Survey of Israel department (govmap.gov.il) at both 2010 and 2019, and were concatenated together to form two large images. The large image of 2010 is shown in Fig E in S1 Appendix. Within this domain 150 locations were chosen manually, each at the center of a small region of 4 stripes of vegetation. This region size of 4 stripes allows us to capture the main properties of the spatial patterns (e.g., rhombic structure), yet small enough to enable us to have many non-overlapping regions. Given that the scale of the trees and their roots are of a few meters (compared to an order of 30x30 [m] for a region), and water flow is also limited (in particular due to the physical modification of the landscape), this means that we this scale is also relevant for the main processes of interest [71]. The choice of locations was based on viewing of aerial images from 2010 alone, and locations were chosen such that they mark the center of regions where stripes are relatively regular in shape, so that they do not overlap, and so that they are spread out throughout the domain.

For each of these 150 regions, an automatic algorithm was then run, to find the best center point of the region (i.e. between the two middle vegetation stripes), and the angle of the stripe. This was manually corrected for 17 regions, where the algorithm partially failed.

All 150 regions were now comparable (despite different size and angle), in that they all represent square-shaped regions of four parallel vegetation stripes aligned in the same direction. At this point they were given a score based on their spectral densities, to assess how regular their vegetation stripes are, and ordered from the most regular to the least one. This score is the ratio between the total intensities (of FFT-image) of the 2 pixels at (*k*_*x*_, *k*_*y*_) of (-4,0) and (4,0), which represent the mode of four stripes in the image, and all the pixels in the FFT-image within a radius of 8, excluding the central pixel (0,0). The 50 lowest-score regions (with least regular stripes) were discarded (shown by black dots in Fig E in S1 Appendix), and the rest of the 100 regions in this new ordering were now analyzed. In particular, images of the regions from 2010 and 2019 were compared to see the change in vegetation that took place within that time frame.

These 100 regions were classified into 5 categories: 1) Regions with a clear transition to a rhombic pattern. 2) Regions with substantial change, with strong marks of a rhombic pattern. 3) Regions with minimal change. 4) Regions with collapse of most vegetation. 5) Regions with unclear change. See S1 Appendix for details of this classification and analysis. The double set of images of these 100 regions (from 2010 and 2019), and its analysis using spectral densities, is also shown in S1 Appendix.

## Supporting information

S1 Appendix Supplementary figures, glossary and analysis details. Further illustrations of self-organization modes, a glossary of technical terms, and more information about the analysis of aerial images. **Fig A**: Instabilities and associated SO modes for vegetation restoration. **Fig B**: Schematic illustration of different vegetation plantation patterns. **Fig C**: Responses to precipitation downshifts under stochastic precipitation using a Gaussian distribution. **Fig D**: Annual precipitation data from the city of Be’er-Sheva, Israel. **Fig E**: Map of case study domain.
(PDF)
