# Supplementary material for: High-integrity human intervention in ecosystems: Tracking self-organization modes
Source: PLoS Comput Biol. 2021 Sep 29;17(9):e1009427. doi: 10.1371/journal.pcbi.1009427 (PMC8504872; doi:10.1371/journal.pcbi.1009427)
Supplement: S1 Appendix — Further illustrations of self-organization modes, a glossary of technical terms, and more information about the analysis of aerial images. Fig A: Instabilities and associated SO modes for vegetation restoration. Fig B: Schematic illustration of different vegetation plantation patterns. Fig C: Responses to precipitation downshifts under stochastic precipitation using a Gaussian distribution. Fig D: Annual precipitation data from the city of Be’er-Sheva, Israel. Fig E: Map of case study domain. (PDF) [file pcbi.1009427.s001.pdf]

Appendix for the article entitled: "High-integrity human intervention in ecosystems: Tracking self-organization modes"

Yuval R. Zelnik[1,2], Yair Mau[3], Moshe Shachak[4], and Ehud Meron[5,6]

[1]Centre for Biodiversity Theory and Modelling, Theoretical and Experimental Ecology Station, CNRS, Moulis, France
[2]Department of Ecology, Swedish University of Agricultural Sciences, Uppsala, Sweden
[3]The Institute of Environmental Sciences, The Robert H. Smith Faculty of Agriculture, Food and Environment, The Hebrew University of Jerusalem, Rehovot Israel
[4]Mitrani Department of Desert Ecology, Blaustein Institutes for Desert Research, Ben-Gurion University of the Negev, Sede Boqer Campus, Israel
[5]Department of Solar Energy and Environmental physics, Blaustein Institutes for Desert Research, Ben-Gurion University of the Negev, Sede Boqer Campus, Israel
[6]Department of Physics, Ben-Gurion University of the Negev, Beer-Sheva, Israel

Abstract

This Appendix gives further illustrations and figures, a glossary of terms, and more information about the analysis of empirical afforestation project (in particular, the full results from the analysis of the 100 regions studied).

1 Illustration of concepts and additional figures

Instabilities for vegetation rehabilitation model

Fig A shows the time development of two instabilities and the spectral densities of the patterns that emerge for the model of vegetation rehabilitation, from which the SO modes can be extracted. The first instability (Fig Aa) destabilizes the bare-soil state as the precipitation rate P is increased beyond a threshold value (see Fig 3a). As the spectral density shown in the rightmost panel indicates, the SO mode associated with this instability is a stripe *Fourier mode* of the form $A(t)\cos(kx+\phi)$ with a wavenumber $k = k_f$ even though the natural wavenumber that the system tends to form, k_0, is generally different from k_f. The growth of this mode directs the system towards a *resonant pattern* of parallel stripes consisting of a vegetation band at each modulation stripe.

The second instability (Fig Ab) destabilizes the resonant stripe pattern as P is decreased below another threshold value (see Fig 3a). The SO modes associated with this instability are a symmetric pair of oblique Fourier modes, $a(t)\cos(k_x x \pm k_y y + \phi_\pm)$, where $k_x = k_f/2$ and k_y is such that the total wavenumber k is equal to the natural

wavenumber k_0, i.e. satisfying $k^2 = k_x^2 + k_y^2 = k_0^2$, as the rightmost panel in Fig Ab shows [1]. The growth of these modes directs the system towards a resonant rhombic pattern (frame $t = 125$ in Fig Ab).

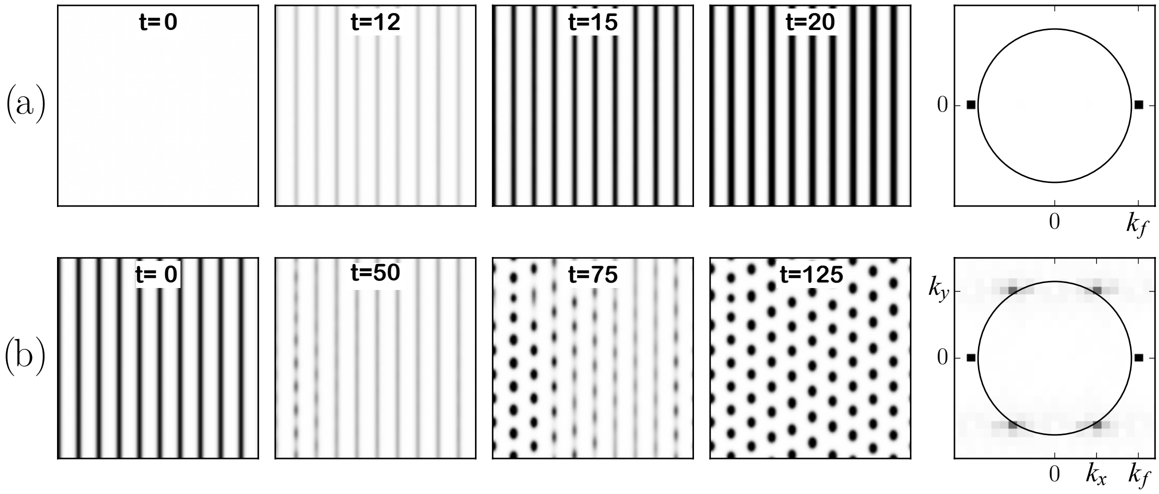

Figure A: **Instabilities and associated SO modes for vegetation restoration** by stripe-like ground modulations. The four panels from left in each row show snapshots of biomass distributions in the (x,y) plane, obtained by solving Eqs (2) and (3) in SI beyond the instability of bare soil to a stripe pattern (a) and the instability of a stripe pattern to a rhombic pattern (b). The rightmost panels show spectral densities of the asymptotic stripe and rhombic patterns in the Fourier plane (k_x, k_y). They reveal the SO modes (dark dots) that grow beyond the instabilities: (a) a resonant stripe mode ($k_x = k_f, k_y = 0$), (b) resonant oblique modes ($k_x = k_f/2, \pm k_y$), lying on a circle of radius k_0, the natural wavenumber in the absence of ground modulations. Note that each mode (k_x, k_y) is accompanied by a conjugate mode $(-k_x, -k_y)$, necessary to guarantee the real-valuedness of the state variables.

Different plantation schemes

Two alternative options for restoring degraded vegetation by stripe-like periodic ground modulations, continuous vs. fragmental vegetation plantation, as Fig B illustrates.

Different modeling of stochastic precipitation

We show here the results for ecosystem response under stochastic fluctuations of precipitation, equivalent to the Fig 5 in the main text, but for a different distribution of precipitation values – a Gaussian distribution (Fig C).

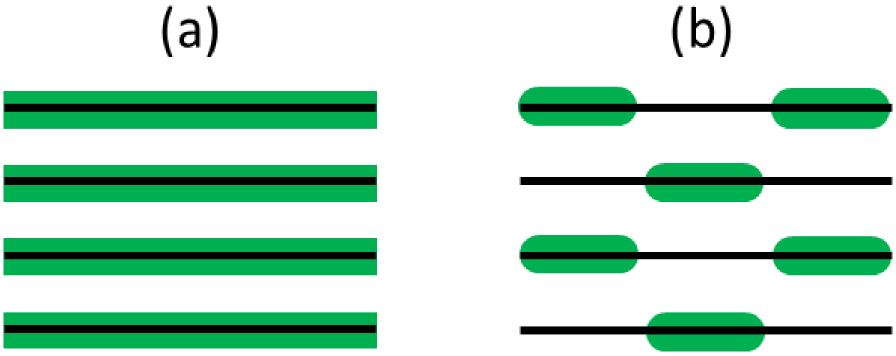

Figure B: **Schematic illustration of different vegetation plantation patterns** (green segments) for a stripe-like ground modulation template (black lines). (a) Continuous plantation to form an overlapping stripe-like pattern, (b) Fragmental plantation to form a rhombic-like pattern.

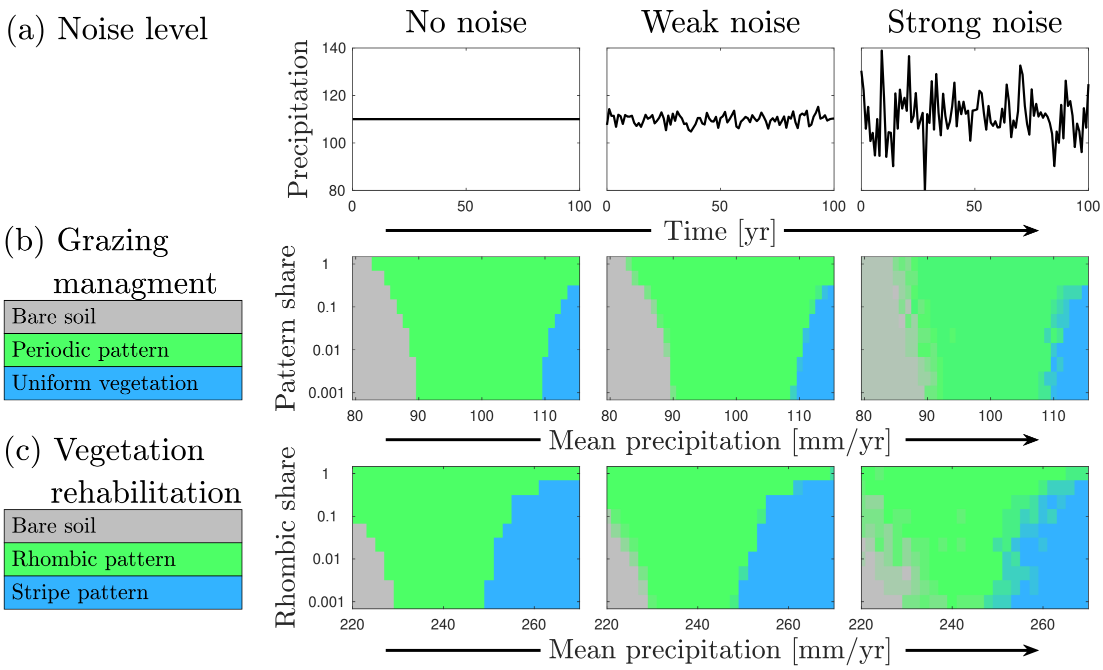

Figure C: **Responses to precipitation downshifts under stochastic precipitation and different initial conditions of mixed vegetation states, using a Gaussian distribution**. This figure corresponds to Fig 5 in the main text, except that the distribution used for creating the precipitation time-series is with a Gaussian distribution rather than a Gamma distribution. Left, middle and right columns correspond to negligible, weak and strong precipitation fluctuations, respectively. **(a)** Demonstration of noise level. **(b)** Asymptotic states (see color legend) for the grazing management system, where initial conditions consist of increasing portions of periodic pattern in uniform vegetation (pattern share). **(c)** Asymptotic states for the vegetation rehabilitation system, where initial conditions consist of increasing portions of rhombic pattern in stripe pattern (rhombic share). Each pixel in the parameter plane (mean precipitation – share) shows the asymptotic state obtained from averaging over 5 randomizations of temporal noise from a Gaussian distribution per simulation, where the initial conditions correspond to mixtures of states calculated at $P = 115$[mm/yr] ($P = 260$[mm/yr]) for middle (bottom) row. Note that this vertical axis is logarithmic.

2 Glossary

Most of the terms appearing below are mathematical concepts. In describing them we favored intuitive clarity over mathematical rigor, given the broad and mostly non-mathematically oriented readership.

Basin of attraction. The set of points in phase space, representing initial conditions that converge in time to the same stable state.

Bifurcation diagram. A diagram that shows the existence ranges and stability properties of possible systems states. The vertical axis represents a state variable (e.g. biomass) while the horizontal axis represents a control parameter. Solid (dashed) lines represent stable (unstable) states.

Growing eigenmode. The direction in phase space along which a system changes following an instability.

More technically, a growing eigenmode is a vector that describes the relationships between state variables that result in the growth of small perturbations as an instability threshold is traversed.

Fourier modes. Functions that describe sinusoidal periodicities in time or space, and are characterized by their frequency or wavenumber values, respectively.

Instabilities of stationary uniform states. Several instability types can be distinguished according to the eigenmode (self-organization mode) that grows beyond the instability threshold:

- *Uniform stationary instability.* An instability that is driven by the monotonic growth of a uniform eigenmode. Such an instability generally results in a different stationary uniform state.

- *Uniform oscillatory instability.* An instability that is driven by the oscillatory growth of a uniform eigenmode. Such an instability leads to uniform time-periodic oscillations (also called 'Hopf instability').

- *Nonuniform stationary instability.* An instability that is driven by the monotonic growth of a spatially periodic eigenmode. Such an instability leads to a stationary periodic pattern (also called 'Turing instability').

- *Nonuniform oscillatory instability.* An instability that is driven by the oscillatory growth of a spatially periodic eigenmode. Such an instability leads to a traveling-wave pattern. (also called 'Turing-Hopf instability').

Instability and bifurcation. A threshold phenomenon in which a combination of feedback processes act in concert to amplify small perturbations about a system state along a particular eigenmode and lead to a state change. A state that lost its stability may still exist as an unstable state. Instabilities are often referred to as bifurcations since the qualitative changes they induce often involve the emergence of new additional states.

Linear stability analysis. A method of pattern-formation theory by which instabilities of system states can be identified by analyzing the dynamics of infinitesimally small perturbations. The analysis provides information about the instability threshold and the feedback processes that drive the instability (through the eigenmode).

Numerical continuation. A method of computing approximate solutions of differential equations by iterative methods and following them as a control parameter is varied. The method is particularly useful for calculating bifurcation diagrams that include unstable solution branches.

Pattern formation theory. The mathematical theory of spatially extended nonlinear dynamical systems, which accounts, among other things, for the spontaneous emergence of spatially non-uniform states in homogeneous systems through symmetry-breaking instabilities. The reader is referred to Ref. [2] for an introduction to pattern-formation theory with applications to ecology.

Phase space (also state space). The space spanned by the state variables of a dynamical system ('phase plane' in the case of two state variables).

Phase-space trajectory. The trajectory emanating from a point in a phase space that describes how the state of a dynamical system changes with time.

Spatial resonance. Self-adjustment of spatial periodicity (wavelength) of a patterned state to an imposed external periodicity. When the external periodicity, λ_f, is close to the system's inherent periodicity, λ_0, the adjustment is 1:1, i.e. the actual system periodicity, λ, is equal to λ_f. When $\lambda_f \approx \lambda_0/2$, the adjustment is 2:1, i.e. $\lambda = 2\lambda_f$.

Resonant pattern. A periodic pattern with a wavelength that matches exactly the wavelength of an externally imposed periodicity.

Saddle point. An unstable system state that has both stable and unstable manifolds, that is, directions of convergence and departure in phase space.

Saddle-node (fold) bifurcation. A collision and disappearance of two states of a dynamical system, as a control parameter traverses a threshold value.

Spectral density. The distribution of power among the Fourier modes of an oscillating state or of a spatial pattern. The power of a Fourier mode is measured by the square of the absolute value of its amplitude. Periodic patterns are characterized by spectral densities with distinct peaks at wave-vectors that describe the pattern's periodicity.

Stable manifold of a system state. The set of points (initial conditions) in phase space that converge at long times to the system state.

Stable state. A system state that recovers from any sufficiently small perturbation or disturbance. The state can be stationary or oscillatory, spatially uniform or periodic.

Stationary state. A state of a dynamical system for which all state variables are independent of time. A stationary state can be spatially uniform, spatially periodic or disordered. The latter two are also called periodic or disordered stationary patterns.

Unstable manifold of a system state. The set of points (initial conditions) in phase space that converge to the system state when time is run backwards. The eigenmode that grows at an instability point provides an approximation to the unstable manifold in the vicinity of the system state.

Unstable state. A system state that evolves towards a different state when subjected to small perturbations of a certain form, e.g. perturbations that contain a particular wavenumber.

Wavenumber. Spatial frequency of a periodic pattern. The wavenumber k of a periodic pattern in a one-dimensional (1d) system is related to the pattern's periodicity or wavelength λ through the relation $k = 2\pi/\lambda$. In 2d systems the wavenumber is the magnitude of the wavevector $k = (k_x, k_y)$, i.e. $k = \sqrt{k_x^2 + k_y^2}$, where $k_x = 2\pi/\lambda_x$, $k_y = 2\pi/\lambda_y$ and λ_x, λ_y represent the periodicities of the pattern in the x, y directions.

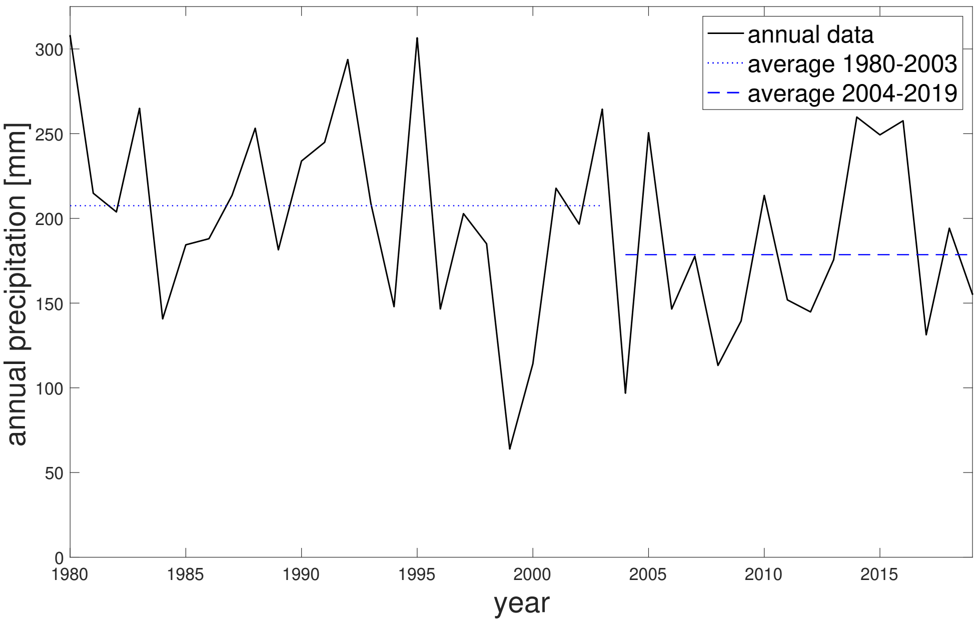

Figure D: **Annual precipitation data from the city of Be'er-Sheva, Israel**, just south of the afforestation area considered. A year is considered to start in the previous August, and end in July of the year, see details in the text. Dotted (dashed) lines show the average precipitation calculated for the years 1980-2003 (2004-2019), which is 207.5[mm] (178.6[mm]).

3 Analysis of empirical afforestation project

3.1 Precipitation data

In order to demonstrate the precipitation regime for the afforestation project in the Northern Nevgev, Israel, we collected precipitation information from Israel's meteorological service (https://ims.data.gov.il). We chose data from the nearby stations in the city of Be'er Sheva (a few kilometers south of the afforestation area, see next subsection) in the time-frame of 1980-2019. Three stations had usable information, in the following years: A) Be'er-Sheva, South (station #251900) during 1980-1995; B) Be'er-Sheva, Negev Institute (station #251690) during 1980-2009; C) Be'er-Sheva (station #251691) during 2004-2019. For each station, we collected monthly precipitation data, and summed over the winter seasons, where we included the first 7 months in a year with the last 5 months of the previous year in a single year (precipitation is negligible between June and August). For instance, the precipitation between, and including, August 2018 to July 2019 was summed and noted as the precipitation for the year 2019.

The annual precipitation of each station and the averaged is given in table 3.1. The averaged precipitation data is also visualized in Fig D, that highlights that the average in 1980-2003 was approximately 205[mm], while it is lower in recent years, with an average of approximately 180[mm] in 2004-2019.

year	Be'er-Sheva, South (station #251900)	Be'er-Sheva, Negev Inst. (station #251690)	Be'er-Sheva (station #251691)	overall average
1980	305.2	310.6		307.9
1981	206.7	223.0		214.8
1982	189.9	217.7		203.8
1983	255.7	274.2		264.9
1984	137.0	144.4		140.7
1985	176.6	192.2		184.4
1986	191.0	185.0		188.0
1987	222.2	205.1		213.7
1988	244.4	262.0		253.2
1989	177.9	185.0		181.4
1990	241.2	226.5		233.9
1991	237.1	252.7		244.9
1992	307.0	280.5		293.8
1993	216.1	201.4		208.7
1994	151.7	144.1		147.9
1995	317.6	295.5		306.5
1996		148.2		146.6
1997		202.8		202.8
1998		185.0		185.0
1999		63.9		63.9
2000		114.2		114.2
2001		217.8		217.8
2002		196.6		196.6
2003		266.1		266.1
2004		45.3	148.4	96.9
2005		260.4	240.7	250.6
2006		142.6	150.4	146.5
2007		181.6	173.7	177.7
2008		115.0	111.4	113.2
2009		141.5	137.4	139.5
2010			213.8	213.6
2011			151.9	151.9
2012			144.8	144.8
2013			175.6	175.6
2014			259.8	259.8
2015			249.3	249.3
2016			257.6	257.6
2017			131.3	131.3
2018			194.2	194.2
2019			155.2	155.2

Table A: **Annual precipitation data from the city of Be'er Sheva, in mm per year**.

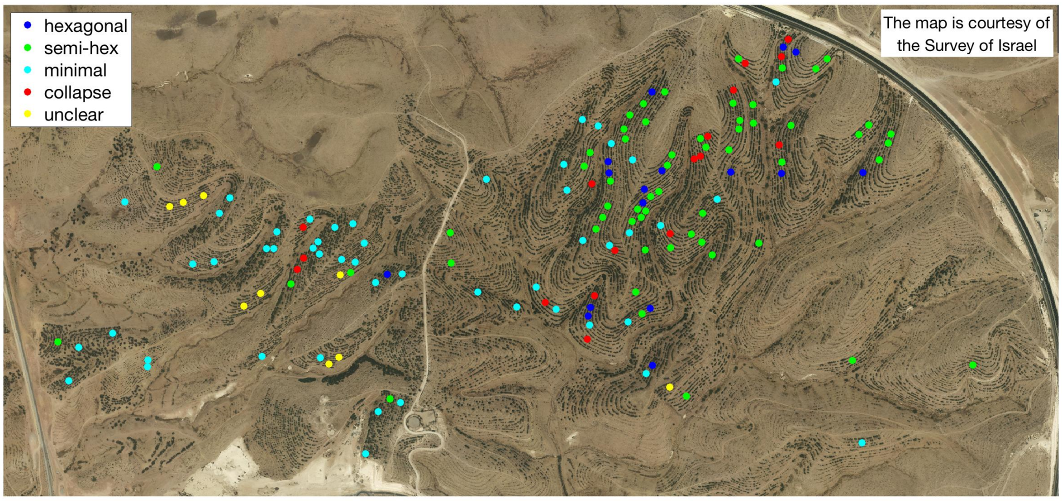

Figure E: **Map of case study domain**, based on aerial images taken in 2010 of a planted forest area. Location is the northern Negev, Israel (Coordinates: 31.295N, 34.815E), in an area of approximately 3.0x1.5 [km]. Colored dots mark locations of 150 regions chosen, each with four stripes of vegetation (color scheme given in legend, and explained in main text).

3.2 Analysis of remote sensing data

We give here further technical details on the analysis and categorization of the aerial images. We also show in Figs F1-F12 results of the analysis for the 100 regions studied.

All regions are located within the area shown in Fig E. The process of choosing the regions, as well as their normalization (i.e. so as each region shows 4 vegetation stripes along the same direction) is described in the main text (Materials and Methods). The data of these regions is given in: https://doi.org/10.5281/zenodo.3902397.

The spectral analysis of the images (initially as RGB true color) was preformed as follows. Each image was converted into a intensity map (grayscale), to facilitate the FFT analysis. This was done using several image processing functions of Matlab. The red channel (of the RGB image) was taken, and the function adapthisteq was used to normalized intensities. The functions imerode and then imreconstruct were used (both with a disk kernel of 5x5), to smooth out the result. This image was then converted to spectral density map using FFT, with the central pixel set to 0 (i.e. ignoring average intensities).

In this way, we now have six representations of each region: a color (RGB) image, a grayscale image, and a spectral density map (i.e. after applying FFT), each for the year 2010 and the year 2019. These six representations are shown, for each of the 100 regions, in Figs F1-F12. For each region, based on the six representation, a manual analysis of the transition between 2010 and 2019 was preformed. The analysis categorized the transition that occurred in each region into one of 5 categories (shown in different colors in Fig E):

- **Category I**: 16 regions show a clear oblique modes (hexagonal pattern in the FFT plane), implying a transition to a rhombic vegetation pattern (Figs F1-F2).

- **Category II**: 48 regions show a substantial change, with some marks of oblique modes (Figs F3-F8).

- **Category III**: 9 regions only show a minimal change in vegetation cover (Fig F9).

- **Category IV**: 9 regions appear to show a collapse of the vegetation, with more than half the trees disappearing (Fig F10).

- **Category V**: 18 regions show a change that is not well detected by the FFT analysis (Fig F11), or are difficult to categorize (Fig F12).

On the top of each region analysis (in the figures below), basic information is given. A score is noted for each region, where a higher score indicates that the 2010 region had a more regular stripe pattern (calculation of region score is defined in the Materials and Methods section of the main text). Besides the score, a region number is given (numbered from highest to lowest score), x&y coordinates (in pixel terms of the image shown in Fig E), its size L (length of the square region), and the angle θ of the region, relative to the image in Fig E. The pixel size (as given in x,y, and L) is 0.165 [m], so that region size ranges between 15[m] and 43 [m].

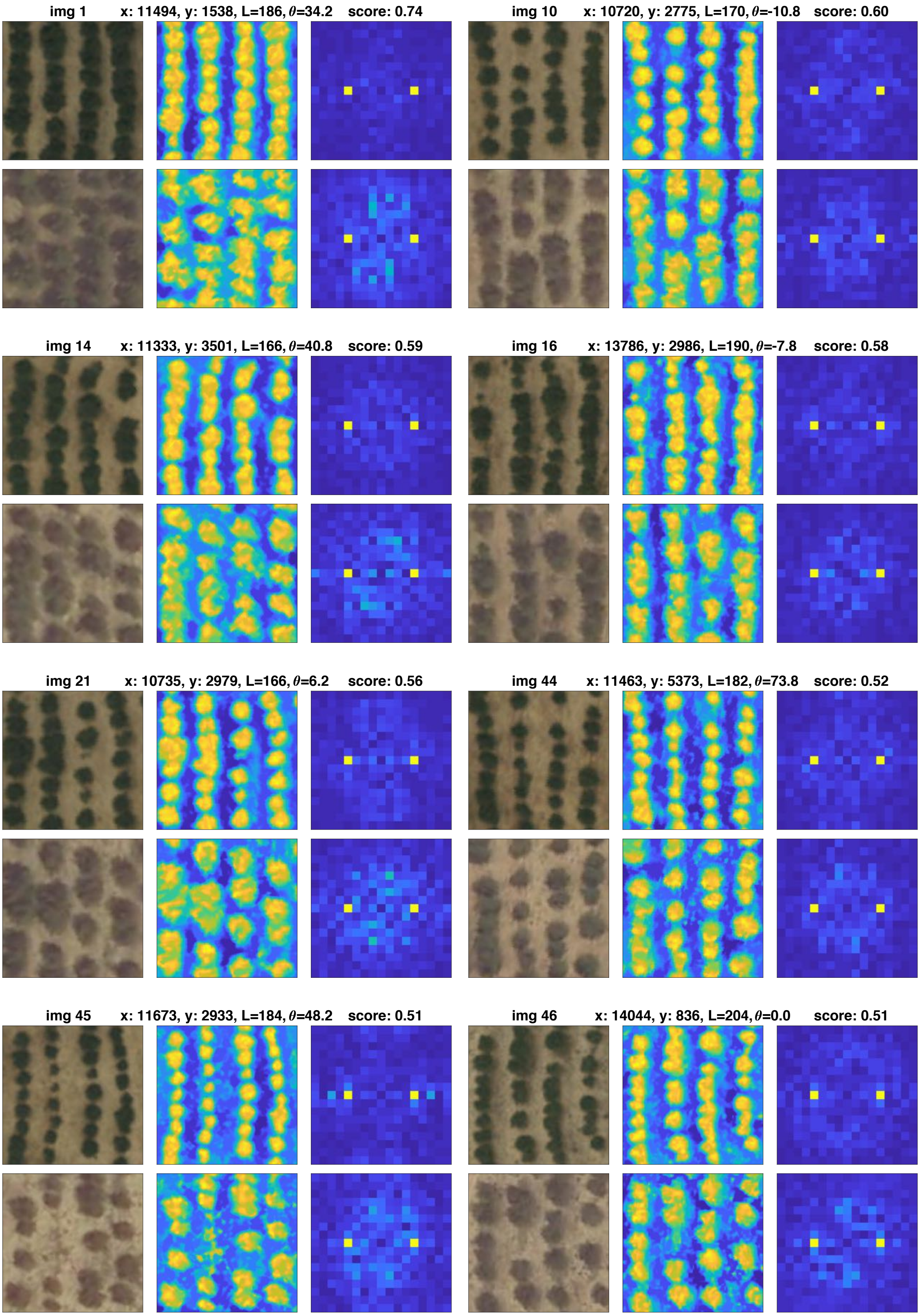

Figure F1: Regions of category I: clear transition to rhombic pattern (hexagonal FFT) – part 1

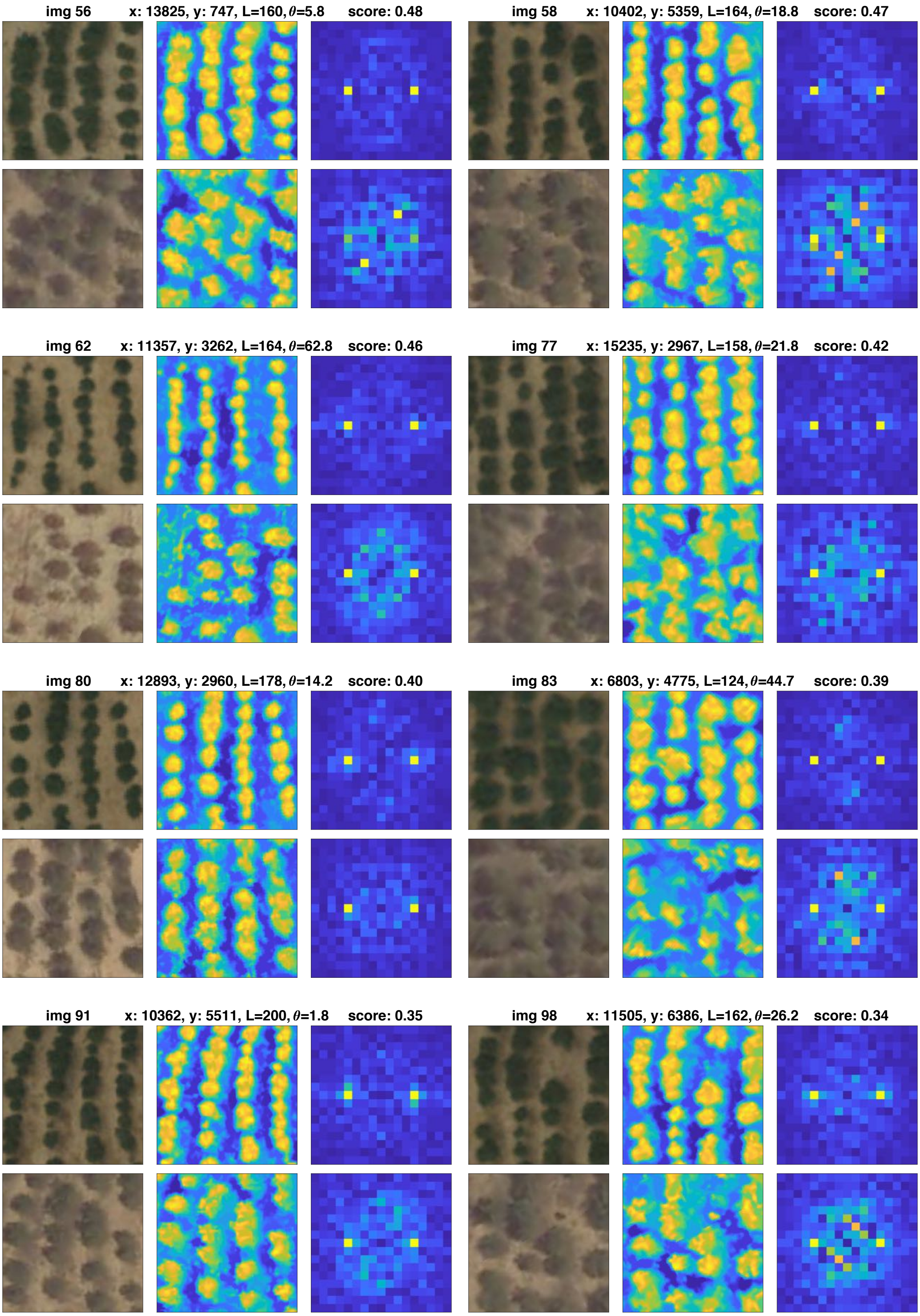

Figure F2: Regions of category I: clear transition to rhombic pattern (hexagonal FFT) – part 2

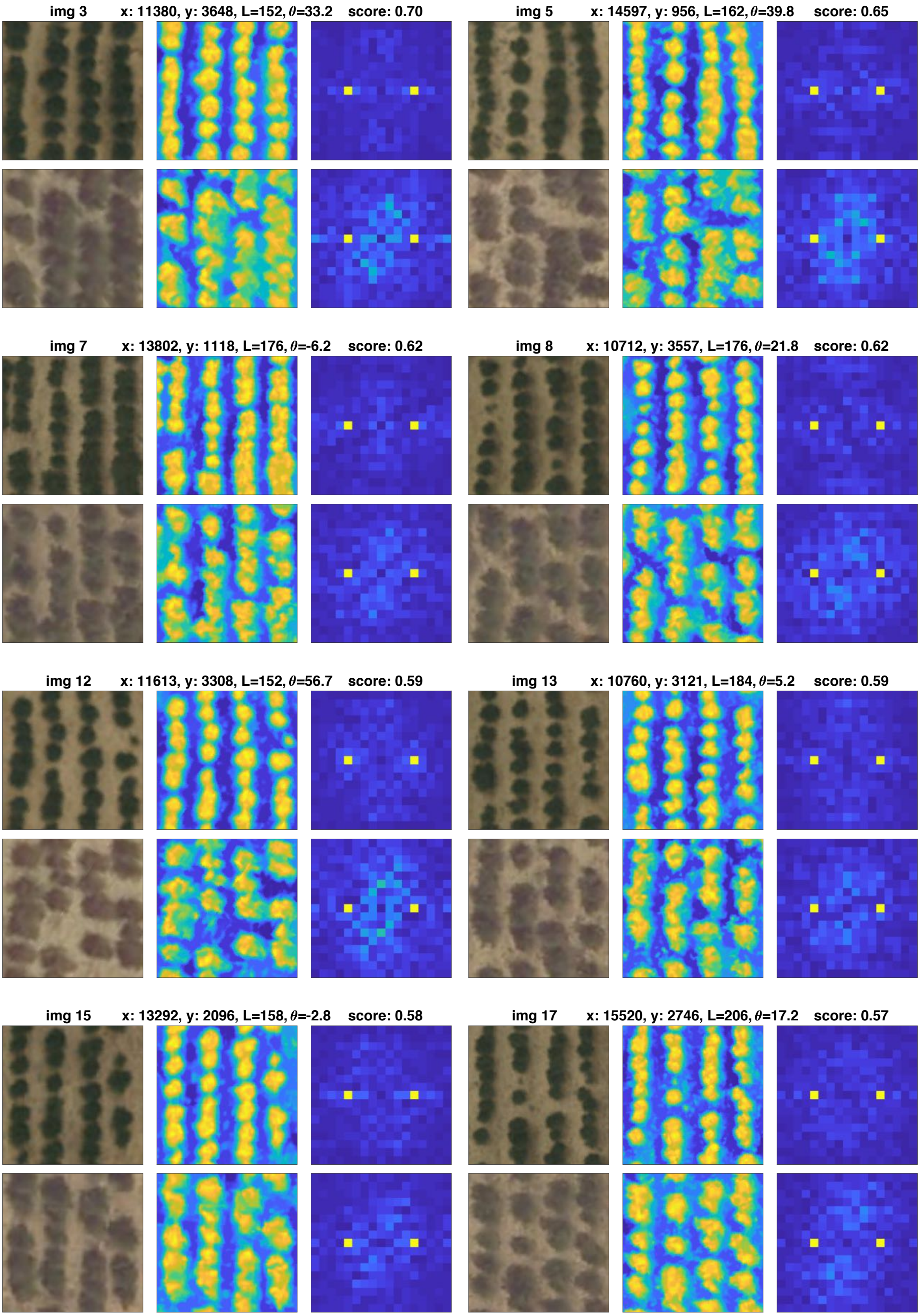

Figure F3: Regions of category II: possible transition to a rhombic pattern - part 1

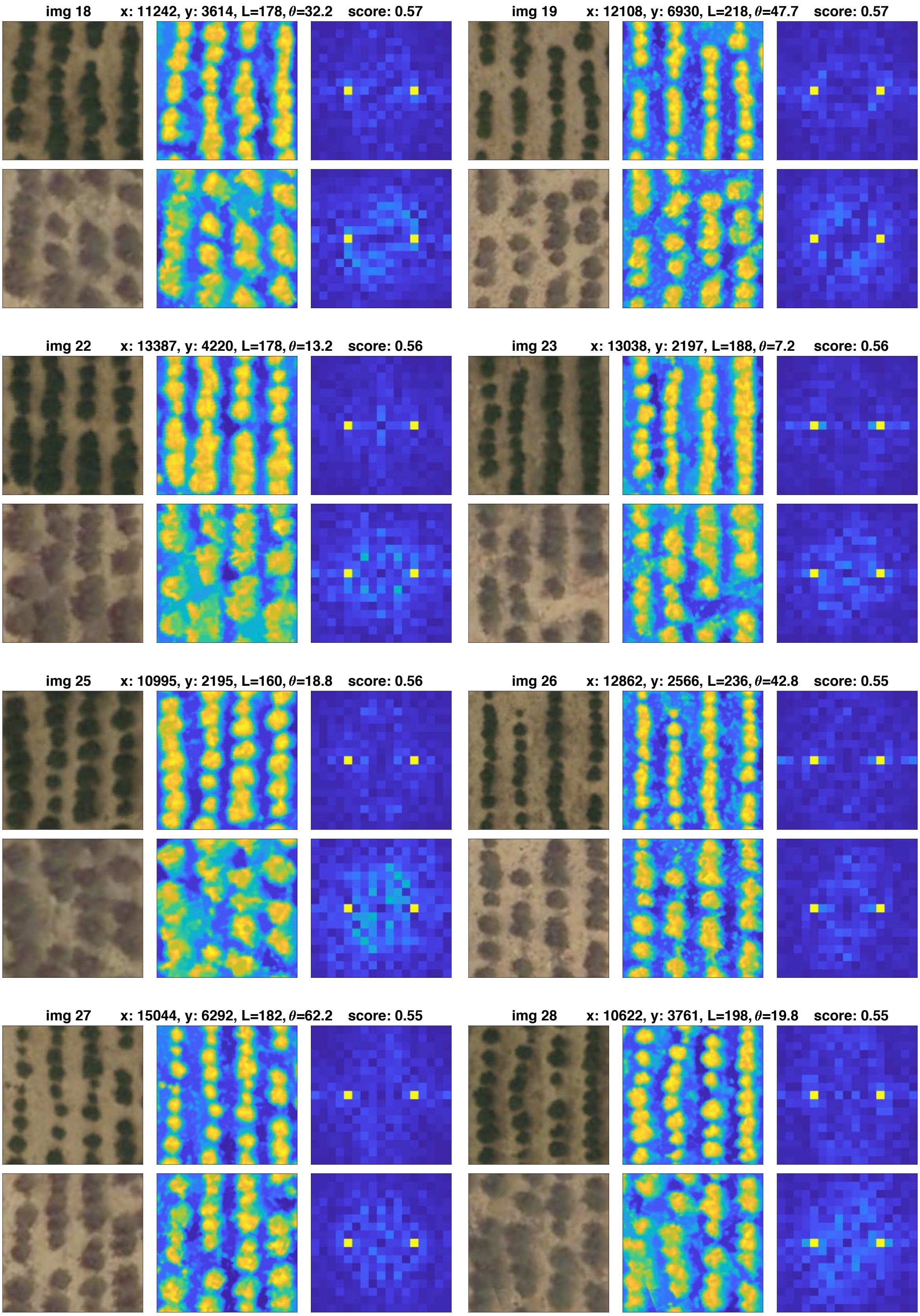

Figure F4: Regions of category II: possible transition to a rhombic pattern - part 2

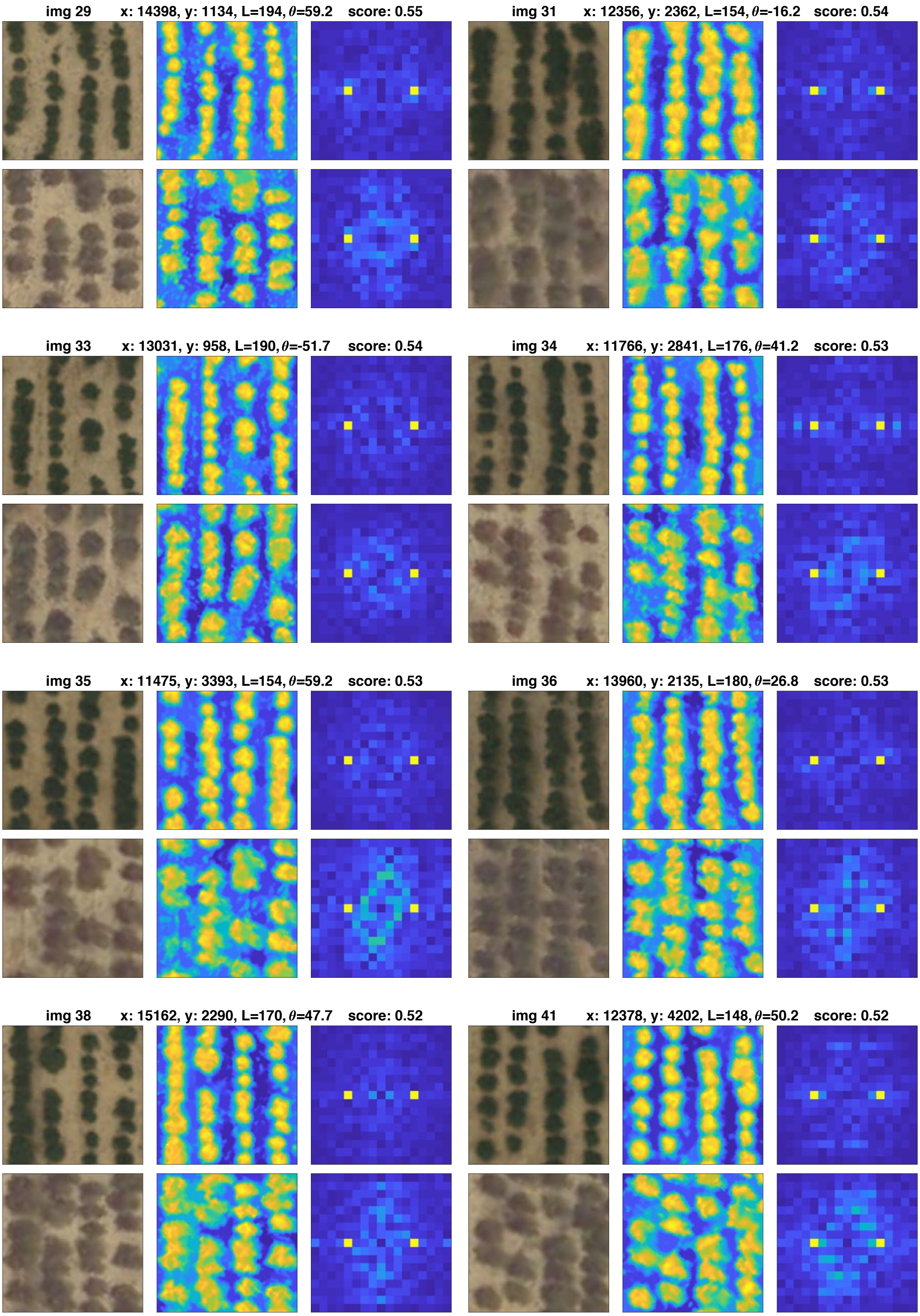

Figure F5: Regions of category II: possible transition to a rhombic pattern - part 3

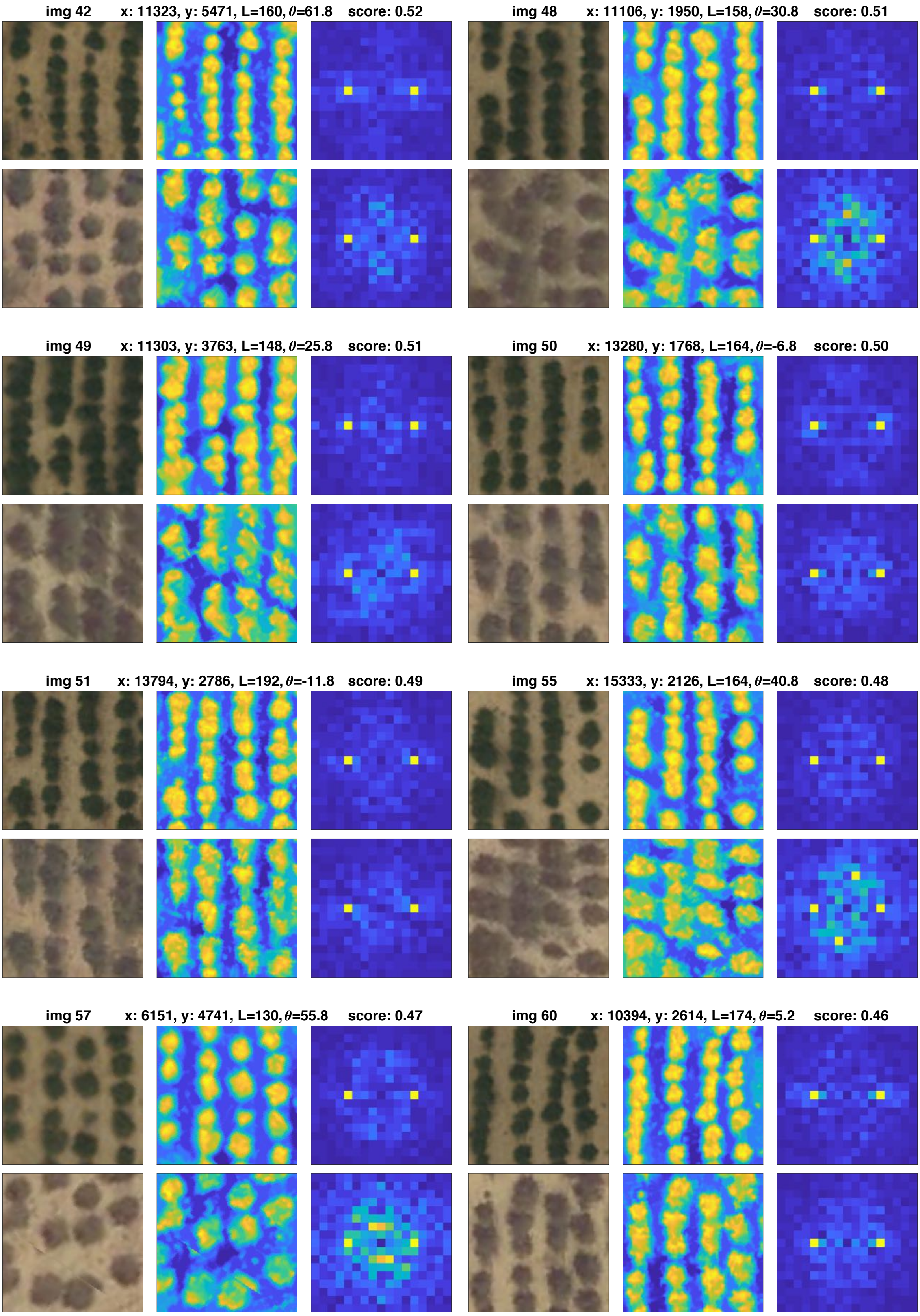

Figure F6: Regions of category II: possible transition to a rhombic pattern - part 4

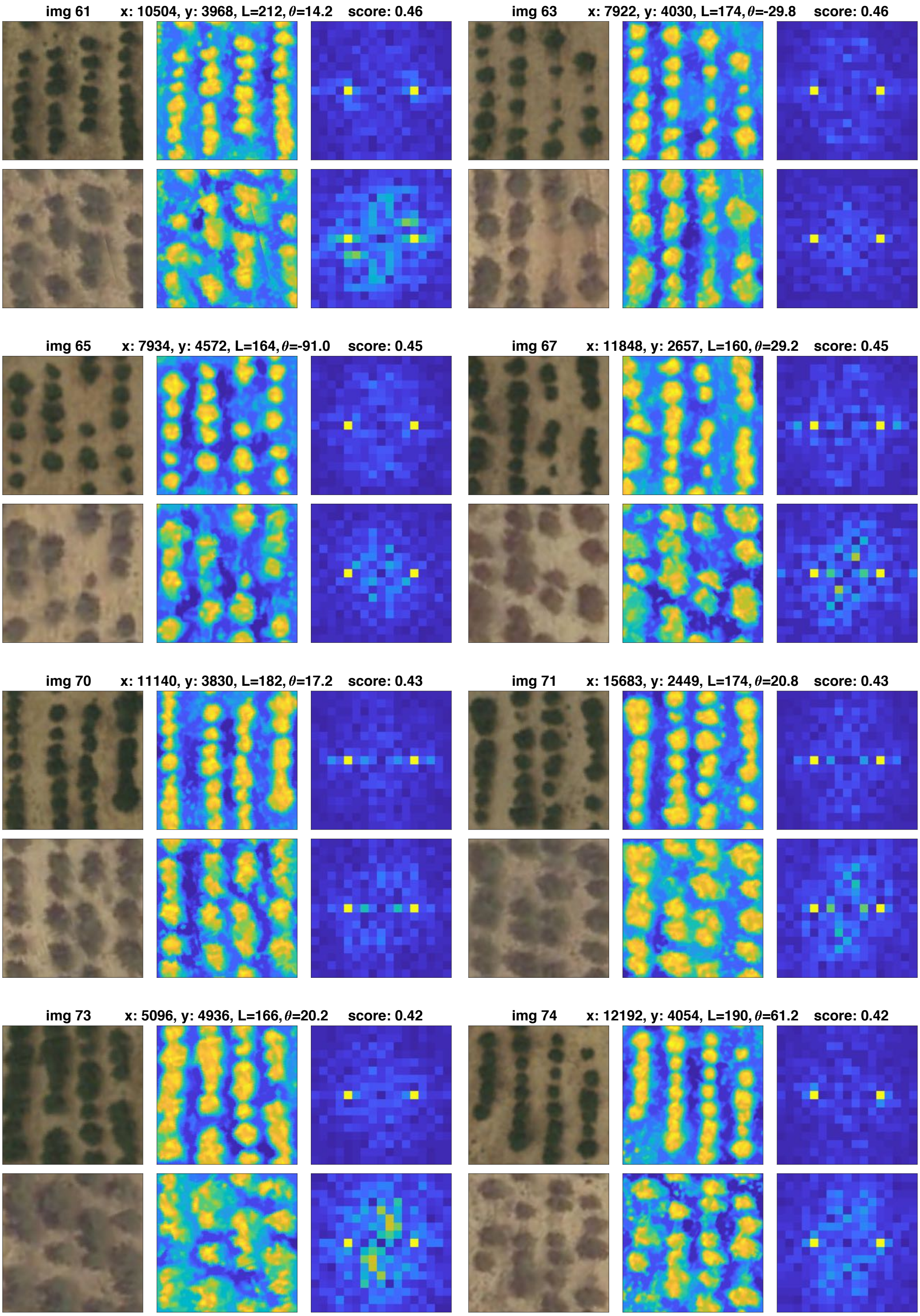

Figure F7: Regions of category II: possible transition to a rhombic pattern - part 5

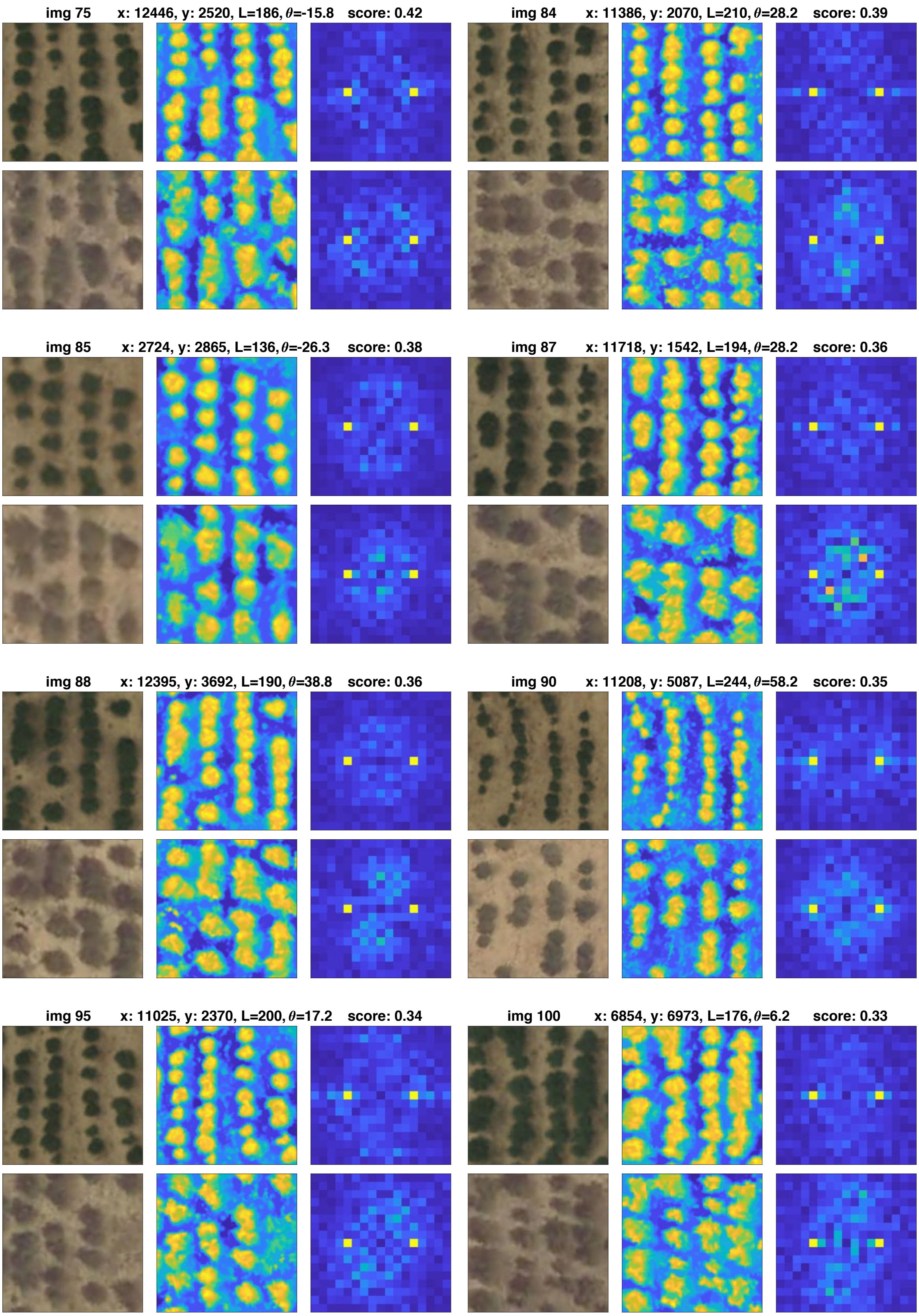

Figure F8: Regions of category II: possible transition to a rhombic pattern - part 6

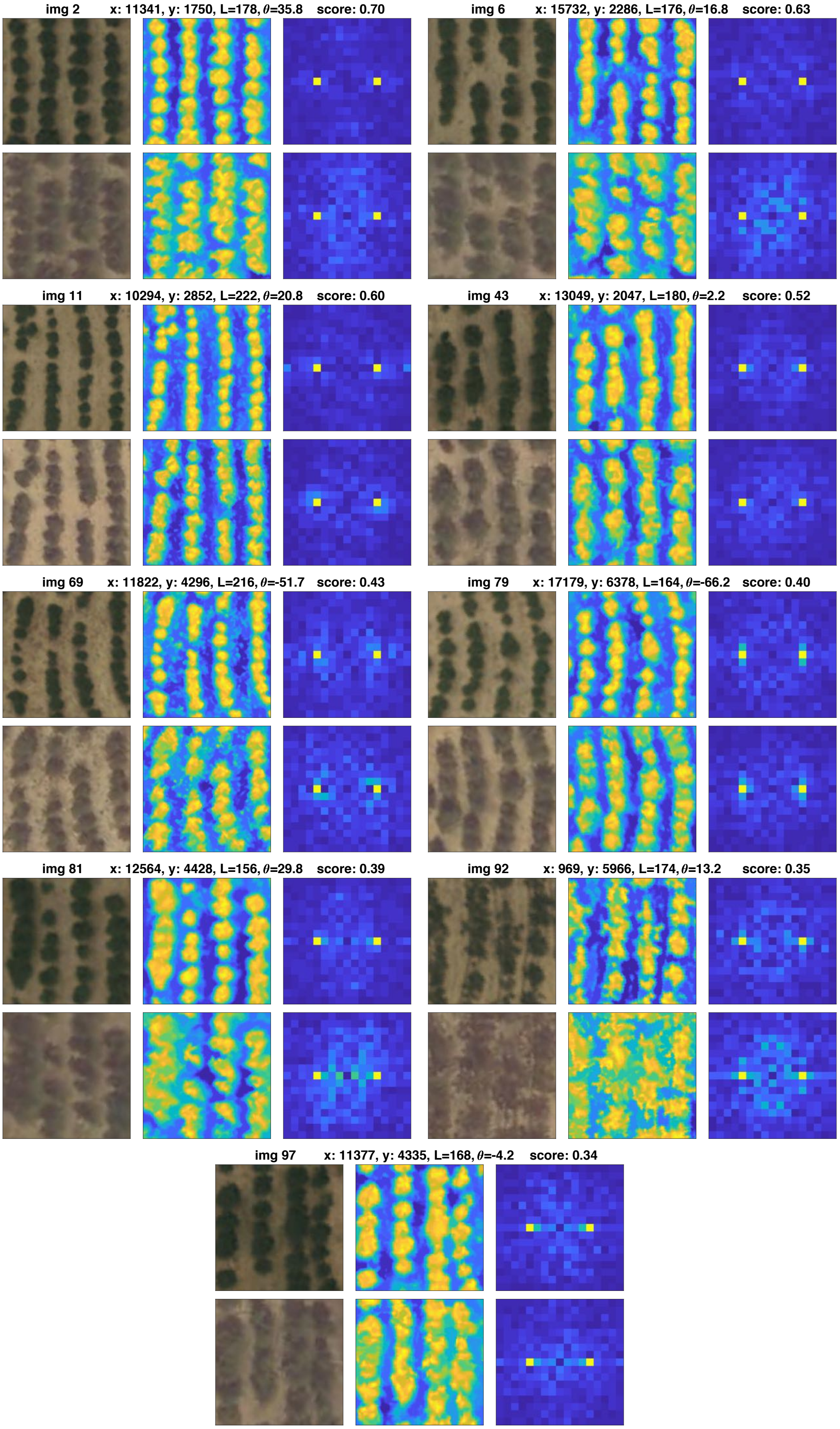

Figure F9: Regions of category III: minimal change of vegetation cover

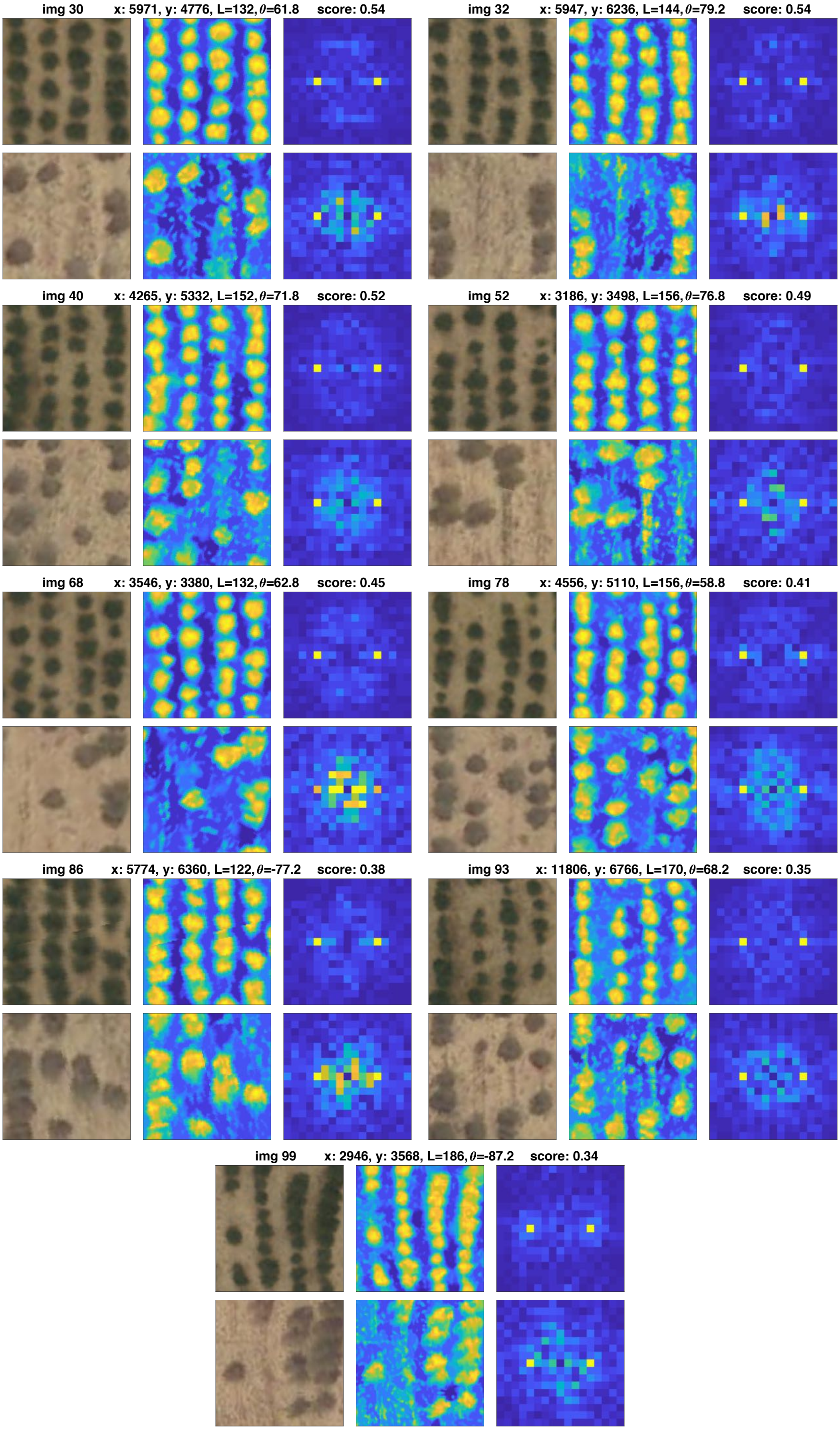

Figure F10: Regions of category VI: Apparent collapse of vegetation

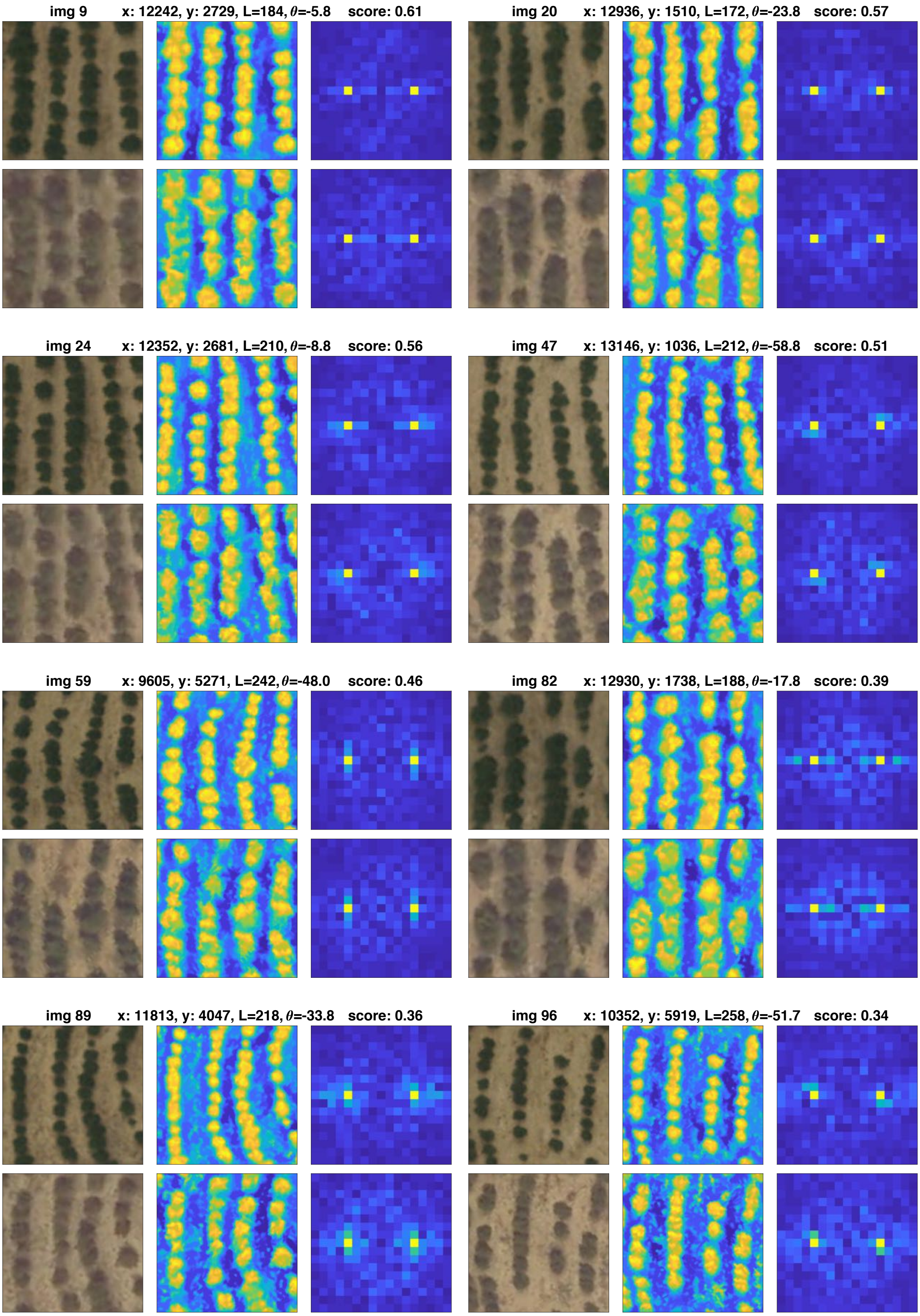

Figure F11: Regions of category V: Unclear change in FFT plane (part 1)

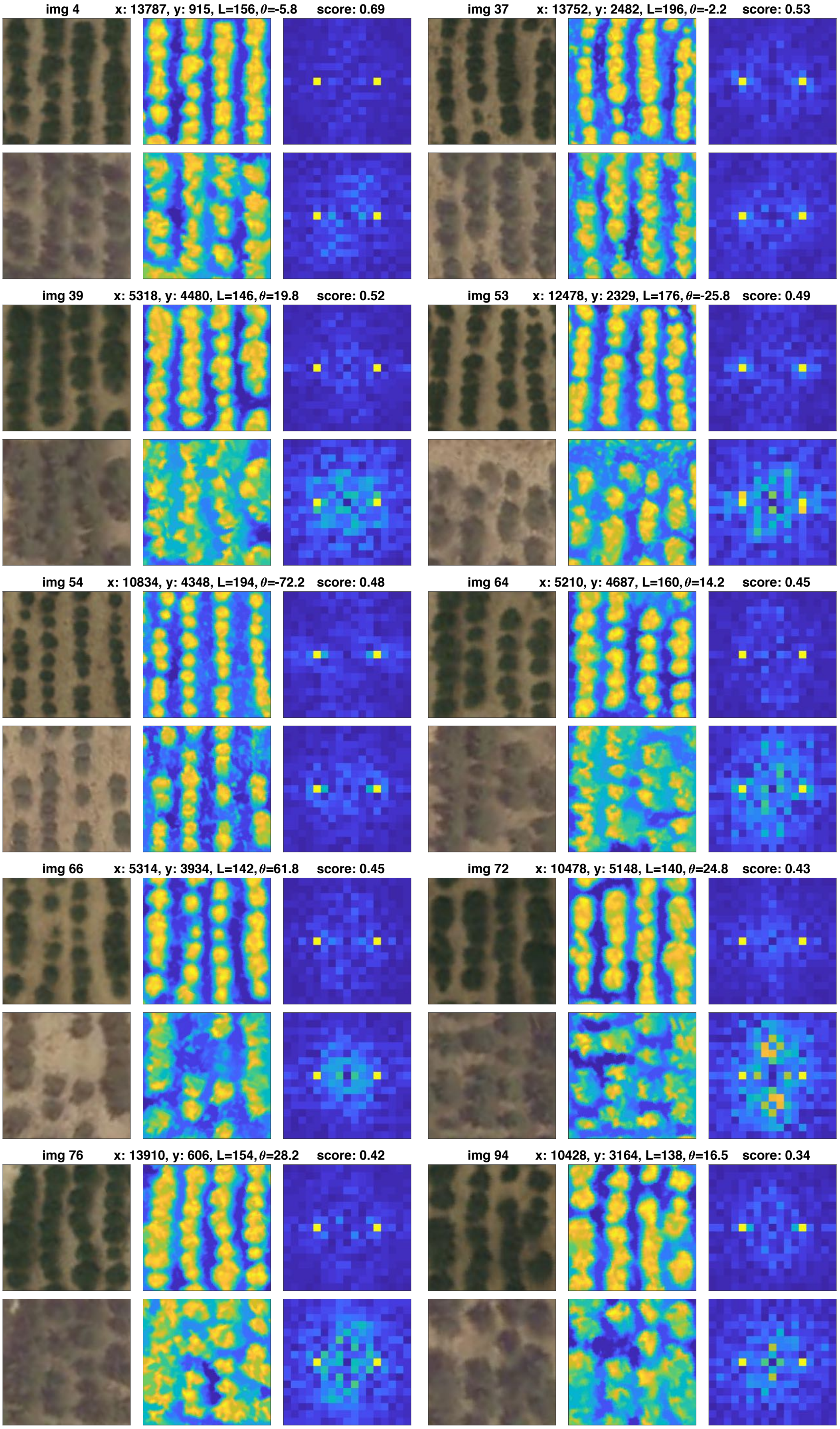

Figure F12: Regions of category V: Difficult to categorize (part 2)

References

[1] Yair Mau, Lev Haim, and Ehud Meron. Reversing desertification as a spatial resonance problem. *Physical Review E*, 91(1):012903, 2015.

[2] Ehud Meron. *Nonlinear physics of ecosystems.* CRC Press, 2015.
